# Proton, Electron, and Hydrogen-Atom Transfer Thermodynamics of the Metal–Organic Framework, Ti-MIL-125, Are Intrinsically Correlated to the Structural Disorder

**DOI:** 10.1021/jacs.5c10498

**Published:** 2025-09-13

**Authors:** Nazmiye Gökçe Altınçekiç, Chance W. Lander, Jiaqi Yu, Ayman Roslend, Yihan Shao, Hyunho Noh

**Affiliations:** † Department of Chemistry and Biochemistry, 6187 The University of Oklahoma, Norman, Oklahoma 73019, United States; ‡ Department of Chemistry, 3270 Northwestern University, Evanston, Illinois 60208, United States

## Abstract

Interfacial charge transfer reactions involving protons and/or electrons are fundamental to heterogeneous catalysis and many other reactions relevant to energy, chemical, and biological sectors. Metal–organic frameworks (MOFs) with redox-active metal-oxo nodes have emerged as candidate materials to examine these reactions with near-atomic-level precision, given their crystalline nature. Here, we employed a colloidally stable, Ti-based MOF, Ti-MIL-125, with different crystal sizes to examine catalytically relevant charge transfer thermodynamics. The Ti_8_(μ_2_-O)_8_(μ_2_-OH)_4_ nodes structurally mimic TiO_2_, which has shown some PCET reactivity toward reactions of H_2_, O_2_, and others. In this report, we have demonstrated that a change in crystal size induces different amounts of structural disorder to the Ti-oxo node, further changing the thermodynamics of proton/electron/hydrogen-atom transfer reactions. Using electrochemical open-circuit potential (*E*_OCP_) measurements, we have determined that all crystallites undergo a 1H^+^/1e^–^ redox reaction, which, given the stoichiometry, can be considered as a net H atom transfer (HAT) reaction. The thermodynamics of this HAT reaction, the Ti^3+^O–H bond dissociation free energy (BDFE), was dependent on the crystal size of the MOF, as the decrease in crystal size induced more structural disorder. Our computational calculations have indicated that this difference in BDFE is due to a local change in the geometry of Ti cations, rather than the commonly invoked defects, such as the “missing-linker” defect sites. Individual proton/electron transfer (PT/ET) thermodynamics were also highly dependent on the crystal sizes. These were probed using p*K*_*a*_ or band gaps (*E*_g_), respectively. These findings suggest that, particularly when MOFs are nanosized with a large amount of structural disorder, they should no longer be considered “true” single-site catalysts; this is an implicit, but widespread assumption within the MOF-based catalysis field. Implications of these findings will be contrasted with structurally similar metal oxides like TiO_2_ and other redox-active MOFs.

## Graphical abstract

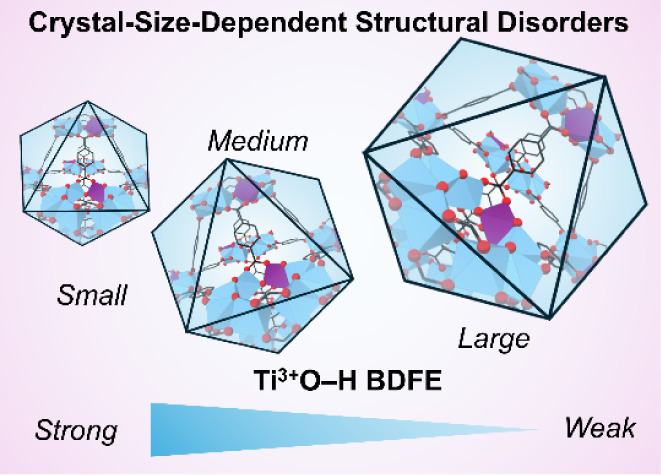

## Introduction

Charge transfer reactions at the interface of a heterogeneous material and surrounding protic liquid often involve protons and/or electrons.^1,2^ Thermodynamics of proton transfers (PT) are typically described using p*K*_*a*_ of surface Brønsted acids, while those of electron transfer (ET) are probed through redox potential (*E*°) or band gap (*E*_g_).^3–7^ Because ET/PT results in a charged intermediate, the two reactions are often strongly coupled as a proton-coupled electron transfer (PCET) reaction involving equimolar amounts of protons and electrons. This PCET reaction is thermochemically related to a H atom transfer (HAT) reaction.^2,8^ In essence, HAT is a “hydrogenation reaction” of a surface binding site; this is illustrated using a generic binary material in Scheme 1 by showing how a homolytic cleavage of H_2_ at the surface also yields the bound H atom. The free energy values of these three reactions are correlated to each other through the formation free energy of an H atom (H•) either from 1H^+^/1e^–^ or 1/2H_2_; these values are typically denoted as C_G_ or Δ*G*°_f_(H•), respectively (eq 1).^9^

**Scheme 1:**
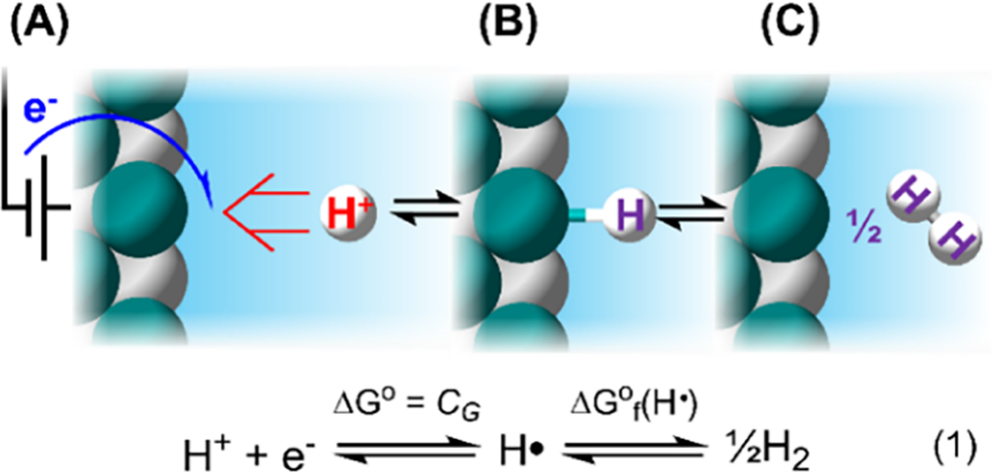
Schematic Illustration of the Thermochemical Equivalence between (A) PCET, (B) HAT, and (C) Hydrogenation of Surface Sites

For many binary semiconductors, including TiO_2_, the electrons involved in the redox reaction are thought to reside in the “trap state.” ^10–13^ A trap state is a type of defect site that is structurally distinct from the bulk. These electrons are typically lower in energy than those in the conduction band (CB), and are thought to be the catalytically responsible sites in reactions of H_2_, CO_2_, and many others.^14,15^ Because these transformations involve protons and electrons, the ET reactions at the trap states must be coupled with protons.^9,16^ Both the proton-to-electron stoichiometry and the thermodynamics of the PCET/HAT reactions at the trap states are intimately correlated to the chemical nature of the trap states, as well as the crystal morphologies, lattice structure, chemical history, and many other factors.^17–22^

The correlation between the exact structure of the trap states and the thermodynamics of ET, PT, and PCET reactions will greatly propel next-generation catalyst discovery relevant to energy and chemical sectors.^20,23^ However, to date, these correlations remain elusive and limited, primarily due to the difficulty in understanding the *exact chemistry* occurring at these structurally disordered sites. Structural disorders are typically dispersed within a bulk lattice structure in a nonperiodic manner. Individual sites may have distinct structures and evolve into different moieties over the reaction period.^24^ Together, these preclude spectroscopic and diffractive characterization to determine the structure. Decades of research in “defect engineering” have significantly enhanced an understanding of how different types of defect sites are critical in catalysis.^25,26^ Still, direct experimental measurements of thermodynamics that govern the reactivity, such as the free energy of PT, ET, and PCET/HAT, and their correlation to the chemical nature of defect sites, are lacking.

Here, we report our findings using the metal–organic framework (MOF), Ti-MIL-125 (Figure 1),^27^ as the model system. We employed this MOF to quantitatively deduce correlations between charge transfer thermodynamics and catalytic sites that are distinct in structure from the bulk lattice. The Ti_8_(μ_2_-O)_8_(μ_2_-OH)_4_ nodes of this MOF can undergo one or two PCET/HAT reactions per node that involve a Ti^4+/3+^ redox reaction, and (de)­protonation at the μ_2_-O site (Scheme 2).^28,29^ This is thermochemically equivalent to Ti^3+^O–H bond formation or cleavage. Thus, we refer to the free energy of this reaction as the Ti^3+^O–H bond dissociation free energy (BDFE).^30^ Scheme 2 highlights this thermochemical equivalence.

**Scheme 2:**
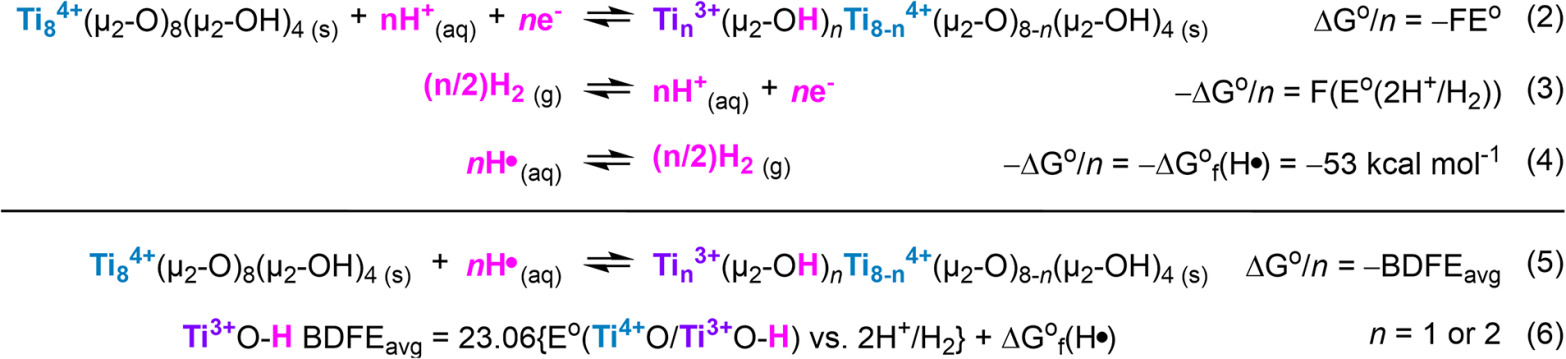
Scheme Illustrating the Thermochemical Equivalence between the Free Energy of PCET Reaction at Ti-oxo Nodes of Ti-MIL-125 and Its Average Ti^3+^O–H Bond Dissociation Free Energy (BDFE)

**Figure 1:**
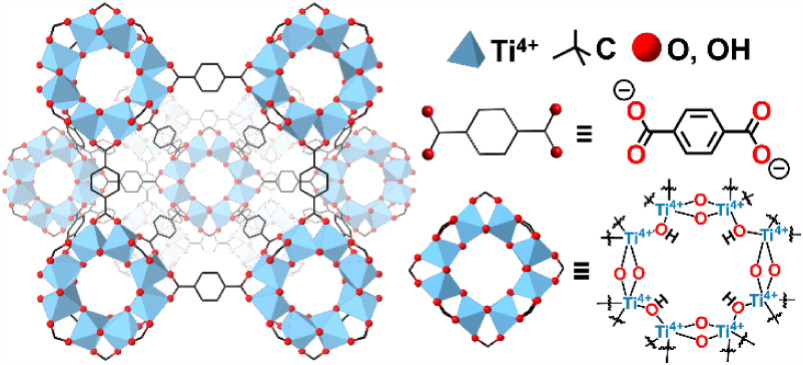
Crystal structure of Ti-MIL-125 and its organic linker and Ti-oxo nodes. This figure was generated from the CIF file reported in ref 27.

This MOF exhibits many unique advantages in assessing the role of any structural disorders in defining the catalytically relevant charge transfer reactions. Fabrizio et al. reported that the synthesis of colloidally stable Ti-MIL-125 with different crystal sizes can be facilely achieved simply by altering the duration of MOF synthesis.^29^ UV–visible spectroscopy can be employed to determine ET thermodynamics. We have previously demonstrated that over a wide range of wavelengths (λ’s), the optical absorbance spectra of Ti-MIL-125 upon reduction linearly scale with its nominal concentration. In other words, the optical absorbance follows the classical Beer–Lambert Law. Notably, the λ values at maximum molar extinction coefficients (ε’s), λ_max_, were highly dependent on the crystallite sizes.^31^ Because these features are due to the d-to-d transition of Ti^3+^,^29^ we concluded that these changes in λ_max_ values indicate geometric distortion or defect sites, primarily at the crystal surfaces; these defect sites may have a distinct coordination environment as to the pseudo-octahedral geometry of Ti^3+^ within a pristine, defect-free node. PT thermodynamics can be determined using potentiometric acid–base titrations, following the reported procedure by Klet et al.^32^

We have further demonstrated recently that the Ti^3+^O–H BDFE of colloidal Ti-MIL-125 can be *experimentally quantified* using a series of open-circuit potential (*E*_OCP_) measurements.^30^ Regardless of the electrolyte composition, the amount of reduced vs oxidized MOF, or pH, the measured *E*_OCP_ values under equilibrium conditions can be predicted by the classical Nernst equation (eq 1). The standard potential derived from the Nernst equation was used to calculate the Ti^3+^O–H BDFE using Scheme 2.

7 $$E_{OCP}=E^{{}^{\circ}}-0.059\log\left(\frac{\left[Ti^{3+}\right]}{\left[Ti^{4+}\right]}\right)-0.059\;pH$$

This report leverages these advantages to correlate ET, PT, and PCET/HAT thermodynamics of Ti-MIL-125 for three different crystallite sizes, and therefore different amounts of structural disorders. As described below, both experimental and computational efforts suggest that all of these thermodynamic parameters are *intrinsically correlated* to the structural disorder but are *independent* of the chemical nature of the surrounding liquid medium. We conclude this report by contrasting the implications of these findings in ET/PT/HAT thermodynamics with other redox-active MOFs and TiO_2_.

## Results

### Synthesis and Characterization of Colloidal Ti-MIL-125 of Different Crystal Sizes

Colloidal Ti-MIL-125 was synthesized according to the reported procedure.^29^ In this work, Ti-MIL-125 crystallites with three distinct sizes were employed. These batches are identical to those used previously in our reports, and much of their characterization can be found in the following references.^30,31^ Many of them are reproduced in the Supporting Information (SI).

The average crystallite sizes were determined using two different approaches: (A) the full-width-half-maximum (fwhm) values of the first three peaks in the powder X-ray diffraction (PXRD) patterns (Figure S2) and (B) the scanning tunneling electron microscopy (STEM) images (Figures S3). These values are shown in Table 1. Each characterization method yielded different crystal sizes, due to assumptions and errors associated with each technique.^33^ Size distributions from STEM images, for example, were systematically larger than those estimated from the PXRD patterns because of the particle agglomeration. However, overall, the three batches of Ti-MIL-125 are distinct from each other. Attempts to measure surface structural disorders using a high-resolution TEM images were unsuccessful, as shown in Figure S4; see further details in the Supporting Information.

**Table 1:** Summary of Average Crystal Sizes of Ti-MIL-125 Used in This Study

	Average sizes of Ti-MIL-125-X (nm) where X =		
Method	S	M	L
PXRD	15	22	33
STEM^a^	19(5)	50(20)	60(20)

a Standard errors represent 1σ of the average (see Figure S3).

N_2_-adsorption–desorption isotherms of the three batches revealed that they are equally porous (Figure S1).

Here onward, these three batches of crystallites will be denoted **Ti-MIL-125-S**, **-M**, or **-L**, (S = small, M = medium, L = large) depending on their crystal sizes.

### Electron Transfer Thermodynamics of Ti-MIL-125

We have previously employed UV–vis spectra to decipher the energy to excite the electron residing in *d*_*xy*_ orbital to the *d*_x2‑y2_ or *d*_z2_ orbitals of Jahn–Teller distorted, pseudo-octahedral Ti^3+^ cations within **Ti-MIL-125-S/-M/-L**.^29^ The energies required for these transitions are listed in Table 2, and details on the measurements can be found in the following reference.^31^

**Table 2:** Summary of *E*_g_, p*K*_*a*_, *E*° vs. RHE, Ti^3+^O–H BDFE of Ti-MIL-125-S, -M, and -L. The Difference in BDFEs, ΔBDFEs, with Respect to Ti-MIL-125-S are Also Shown

		*d*-to-*d* Energy (eV)^b^					
Ti-MIL-125-X	*E*_g_ (eV)^a^	*d*_*xy*_ to *d*_x2‑y2_	*d*_*xy*_ to *d*_z2_	p*K*_*a,A*_/p*K*_*a,B*_ ^a^	*E*° vs RHE (V)^c^	Ti^3+^O–H BDFE (kcal mol^–1^)^d^	ΔBDFE (kcal mol^–1^)^e^
S	3.95(1)	2.07	2.56	1.91(6)/3.5(1)	0.651(5)	68(2)	0
M	3.82(3)	2.02	2.46	1.81(1)/3.8(1)	0.59(2)	67(2)	–1.6(5)
L	3.71(2)	2.00	2.37	1.80(1)/3.78(2)	0.54(3)	65(2)	–2.5(7)

a 1σ reported here are from the average of at least duplicate measurements.

b These energies are from our previous publication (see ref 31).

c *E*° vs RHE values were calculated from Figure 2D by adding 0.059 × pH for each data point in the Pourbaix diagram. The standard error represents 1σ from the average of all data points.

d BDFEs were derived using *E*° vs RHE and eq 6. The standard error of ± 2 kcal mol^–1^ is from ΔGf­(H•), which is larger than 1σ of *E*° vs RHE.

e This is why ΔBDFE values have smaller standard errors than Ti^3+^O–H BDFE (see the Discussion section for more details).

Beyond *d*-to-*d* transitions, UV–vis spectra of the colloidal suspension of pristine MOF were employed to estimate the *E*_g_ values of Ti-MIL-125 (Figure S7); details on experimental protocol and *E*_g_ calculations can be found in the SI. ^1^H NMR of the digested MOF solution in ca. 1 M NaOD was used to determine the rough concentrations of Ti-MIL-125 (see Figure S8). The Tauc plots in Figure 2A demonstrate that as the crystal size decreases, the *E*_g_ value increased by ∼100 meV increments; these *E*_g_ values are similar to those reported by Brozek and coworkers previously^29,34^ and are reported in Table 2. We note that this *E*_g_ is relevant to the photoexcitation of electrons in the highest occupied molecular orbital (HOMO) centered on the terephthalate linker to the lowest unoccupied molecular orbital (LUMO) centered on the Ti cation (*d*_*xy*_); see the report by Hendon et al. for more details.^6^

**Figure 2:**
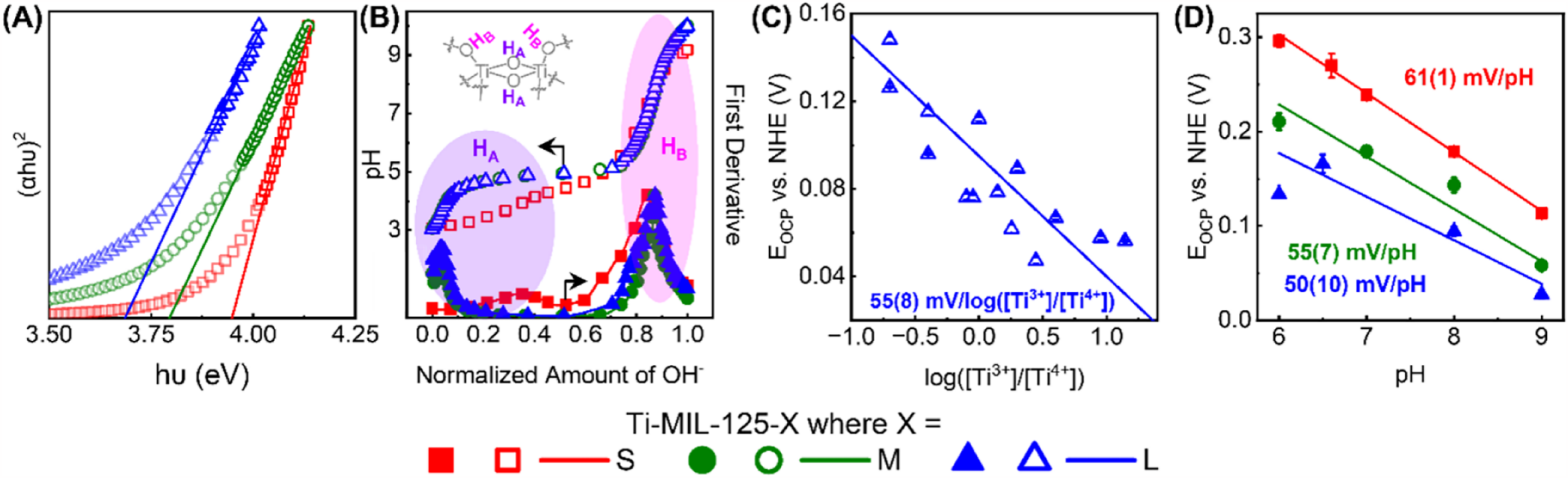
(A) Tauc plots and (B) acid–base titration curves of **Ti-MIL-125-S**, **-M**, and **-L**. (C) The plot of *E*_OCP_ vs log­([Ti^3+^]/[Ti^4+^]) in pH 8-adjusted Tris buffer using **Ti-MIL-125-L**. (C) The plots of *E*_OCP_ vs pH of **Ti-MIL-125-S**, **-M**, and **-L**. (D) The plot of *E*_OCP_ at log­([Ti^3+^]/[Ti^4+^]) = 0 vs electrolyte pH. (C) shows experimental results from triplicate measurements, with different concentrations of Ti^3+^ and Ti^4+^; the error bars, which are all smaller than the size of the data points, represent 1σ of the average *E*_OCP_ at the last 60 s of measurements (see the Supporting Information for details). The error bars in (D) represent 1σ from the linear regressions of (C) and other related plots in the Supporting Information. Standard errors on linear slopes shown in (C) and (D) are 1σ from linear regressions.

### Proton Transfer Thermodynamics of Ti-MIL-125

Beyond the energy of electronic transitions, here, we measured the thermodynamics of PT through potentiometric acid–base titrations. The experimental protocol generally followed that reported previously,^32^ and details are outlined in the Supporting Information.

Figure 2B illustrates how the pH of the solution containing **Ti-MIL-125-S/-M/-L** (originally adjusted to ∼3) changed with sequential titrations of OH^–^. The *x*-axes of all titration curves shown in Figure 2B were normalized to the final amount of the OH^–^ added, as this depended on the exact amount of MOF in the suspension. First-order derivatives of the titration curves indicate that there are two equivalence points per MOF. These equivalence points were used to derive the two p*K*_*a*_ values, denoted p*K*_*a*,A_ and p*K*_*a*,B_, in Table 2. Notably, the titration curves and the derived p*K*_*a*_ values of **Ti-MIL-125-S** were quite distinct from those of **Ti-MIL-125-M** and **-L**. The second p*K*_*a*_ value (p*K*_*a*,B_) is ascribed to the μ_2_–OH within the Ti_8_-oxo nodes (labeled H_B_ in Figure 2B), albeit these values, regardless of the crystal size, are at least one p*K*_*a*_ unit lower than that reported previously.^35^ The other, more acidic p*K*_*a*_ value, p*K*_*a*,A_, is ascribed to the proton on the other eight μ_2_–O moieties between the two Ti cations, which are otherwise deprotonated to maintain the charge neutrality of the node (labeled H_A_ in Figure 2B).

Implications of differences in p*K*_*a*_ and electronic transition energies are further elaborated in the Discussion section.

### Surface Charges and Crystal Sizes of Colloidal Ti-MIL-125 in Electrolytes

This section describes the dynamic light scattering (DLS) and zeta-potential (*E*(ζ)) measurements of colloidal Ti-MIL-125 with three different crystal sizes. All measurements were performed in the buffers and pHs employed in the electrochemical studies, following this section. These are 2-(*N*-morpholino) ethanesulfonic acid (MES), tris­(hydroxymethyl)­aminomethane (Tris), and boric acid (H_3_BO_3_). Concentrations of buffers were kept at 100 mM, and pH values of these electrolytes were adjusted between 6 to 9.

DLS is commonly employed to understand the crystal sizes of colloidal suspensions in an exact liquid medium of reaction. Our measurements indicate that, indeed, **Ti-MIL-125-S** is smaller in size than those of **Ti-MIL-125-M/-L** (Figures S5 and S6). However, overall, the crystal sizes were much larger than those derived from the PXRD patterns or the STEM images (see Table 1). In almost all electrolytes, *E*(ζ) values were negative regardless of the crystal sizes (Figure S9); this corroborates the deprotonation of μ_2_–OH moieties on the Ti-oxo nodes beyond their p*K*_*a*_ values of 3.4 to 3.8 (*vide supra*). In pH 7-adjusted Tris buffer, however, all E­(ζ) values were close to zero as the protonated, and therefore cationic, [TrisH]^+^ can electrostatically interact with the deprotonated nodes. DLS measurements further suggest that in Tris buffers, the apparent crystal sizes were larger (Figures S5 and S6). In fact, **Ti-MIL-125-L** visibly sedimented rapidly in pH 7-adjusted aqueous electrolytes, precluding accurate DLS measurements. As described below, long-term colloidal stability is necessary for accurate *E*_OCP_ measurements, and thus, for **Ti-MIL-125-L**, this electrolyte was omitted for further analysis.

### *E*_OCP_ Measurements of Ti-MIL-125

*E*_OCP_ measurements require the preparation of a reduced Ti^3+^-MIL-125. This was prepared through photoreduction in neat methanol under UV irradiation, as described in the following references.^29–31^

We have previously reported *E*_OCP_ measurements of **Ti-MIL-125-S**.^30^ Here, similar experiments were conducted using **Ti-MIL-125-M** and **-L**. All solutions used in this report were degassed under N_2_ bubbling, and experiments were performed under an N_2_ atmosphere using a standard Schlenk line. Into an aqueous electrolyte, a known volume of pristine **Ti** ^**4+**^ **-MIL-125-M/-L** suspension in methanol was injected. Subsequently, the MOF suspension was photoreduced under UV irradiation and titrated multiple times to change the Ti^3+^ vs Ti^4+^ ratio. After each titration, *E*_OCP_ was measured until the value stabilized with a change of <5 mV min^–1^. Here onward, we focus on measurements performed in pH 8-adjusted Tris buffer. In this electrolyte, all crystallite sizes retained long-term colloidal stability (see above for more details). We have previously found that the concentrations of buffers, the nature of the electrode, and the inevitable addition of oxidized products of methanol, like formaldehyde, do not affect the thermochemical measurements.^30^ Details on experimental protocol and results using other buffers can be found in the SI.

Upon titration of **Ti** ^**3+**^ **-MIL-125-M/-L**, the *E*_OCP_ changed drastically and then equilibrated after ∼300–1000 s; Figures S10–S17 show the *E*_OCP_ vs time trace for all electrolytes. This behavior is very similar to that observed using **Ti-MIL-125-S**.^30^ The *E*_OCP_ values at equilibrium were plotted against the log­([Ti^3+^]/[Ti^4+^]). Representative results using **Ti-MIL-125-L** in pH 8-adjusted Tris buffer are shown in Figure 2C. For results using other crystal sizes and electrolytes, see the SI. Regardless of the crystal sizes and over more than 2 orders of magnitude of a change in concentration ratio, all linear fits have slopes that are close to ∼59 mV per log­([Ti^3+^]/[Ti^4+^]). Fitting the observed *E*_OCP_ values to the Nernst equation shown above (eq 1) suggests that one electron was transferred during the redox reaction for all crystallites.^36^

Formal potentials (*E*°’) are defined as the electrochemical potentials at which the concentrations of the two redox states are identical—i.e., log­([Ti^3+^]/[Ti^4+^]) = 0. Plotting *E*°’ values against the pH values of the electrolytes resulted in Pourbaix diagrams (Figure 2D). Again, the “Pourbaix slopes” based on the linear fits were close to 59 mV/pH for all three crystallites. According to the Nernst equation, this suggests equal stoichiometry between the electron and the proton transferred during the redox reaction. In other words, regardless of the crystallite sizes, Ti-MIL-125 undergoes a 1H^+^/1e^–^ redox reaction.^4,37–40^

### *E*° vs 2H^+^/H_2_ and Ti^3+^O–H BDFE of Ti-MIL-125

The PCET thermodynamics can be expressed in standard potential (*E*°) referenced against the 2H^+^/H_2_ couple in a given proton activity. In aqueous electrolytes, this is the reversible hydrogen electrode (RHE). As shown in Scheme 2, this essentially is equivalent to converting the electrochemical potential of a PCET reaction to that of a hydrogenation reaction. This allows direct comparison of thermodynamics between different systems, pHs, and buffers.^9,38,41^ These values for all crystallites are reported in Table 2.

Derivation of Ti^3+^O–H BDFE requires an additional constant, the formation free energy of H• from H_2_ , Δ*G*°_f_(H•·); this is roughly 53 kcal mol^–1^ in water and in nearly all other solvents.^9,40^ Together, the Ti^3+^O–H BDFE of **Ti-MIL-125-S, -M,** and **-L** were 68(2), 67(2), and 65(2) kcal mol^–1^, respectively; see Table 2. Notably, as the crystal size decreases, the BDFE increases. As discussed later, the ± 2 kcal mol^–1^ error that seemingly questions the difference in BDFE is largely irrelevant when the BDFEs are compared to each other.

### Computational Calculations of Ti^3+^O–H BDFE at Defect Sites

“Missing-linker” defect sites are one of the most commonly invoked types of defect sites on Ti-MIL-125.^42–44^ Computational calculations were performed on these defect sites to examine their PCET thermodynamics. Here, we employed the PBE0 functional^45^ with D3­(BJ) dispersion and dampening^46,47^ and the def2-SVP basis set in Q-Chem 6.3,^48^ following our previous publication.^30^ All organic linkers were displaced by formate linkers.^49,50^ Geometric optimization was performed using the partitioned rational-function optimization (R-PFO) reported by Baker.^51^ Using the geometric direct minimization algorithm (GDM), the self-consistent field (SCF) energy was converged to a cutoff of 1 × 10^–6^ au^52^ As described previously, entropic components were not introduced due to the arbitrarily high translational entropy of an unbound H atom in vacuum.^30^ Thus, all calculated thermodynamics should be considered as bond dissociation energy (BDE). We note, however, that entropic components are usually small for HAT reactions, and thus BDEs are nearly similar to BDFEs (cf.^9^). The XYZ coordinates are available in the Supporting Information.

We first focus on defect sites with an additional 1H^+^/1e^–^. The geometry-optimized structure of the node with one missing-linker defect site is shown in Figure 3A. The extra proton can reside on the bridging μ_2_–O, or the newly introduced terminal −OH, while the electrons reside on the Ti cations. As shown in Figure 3B and C, the added proton and electron were localized, much like those on defect-free nodes (see ref 29, 30 ). Their Ti^3+^O–H BD­(F)­E values, however, were lower than any of the experimentally derived values or from computed values of the pristine node; the two structures have BDFEs of 51.0 or 58.9 kcal mol^–1^.

**Figure 3:**
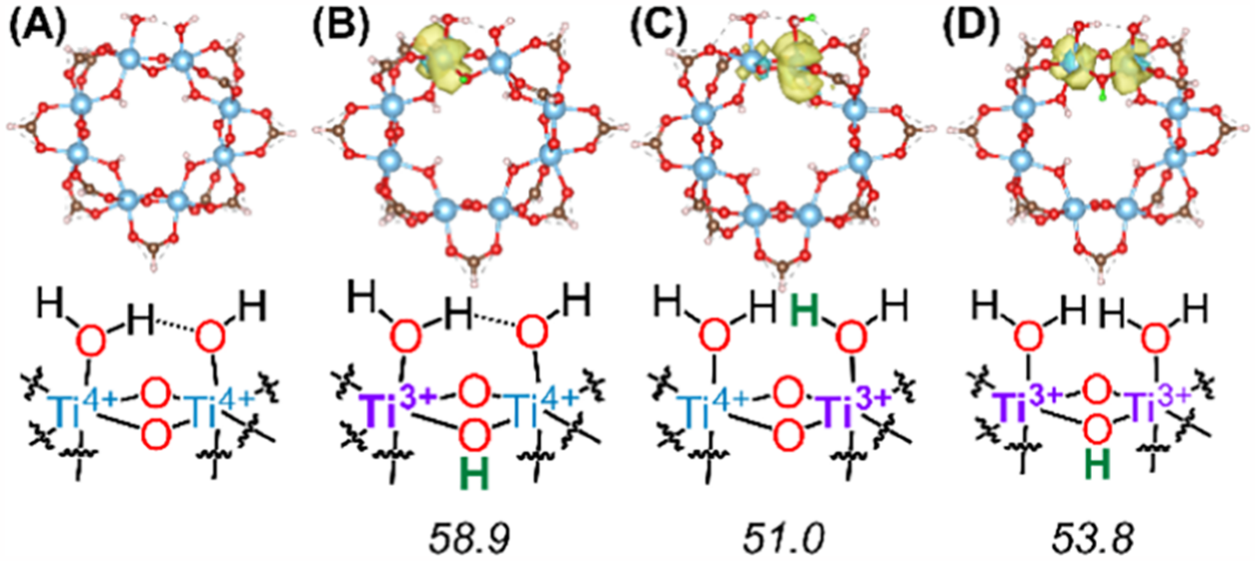
Computationally modeled structures of Ti-MIL-125 nodes with a missing-linker defect site. Geometry-optimized structures of a missing-linker node with no H^+^/e^–^ are shown in (A). (B–C) are nodes with 1H^+^/1e^–^ added to (A). (D) is a node with 2H^+^/2e^–^ on a missing-linker defect site. Schematic illustrations of the relevant portion of the nodes are shown below each figure. For (B–-D), calculated BDE values in kcal mol^–1^ are also shown at the bottom. Bond distances can be found in the SI. Spin density colors: spin up = yellow, spin down = blue. Atom colors: added H atoms = green, Ti = blue, O = red, C = brown, H = white.

Given that a Ti_8_ node within Ti-MIL-125 can accept up to 2H^+^/2e^–^,^30^ we have introduced additional 1H^+^/1e^–^ to the structures shown in Figure 3B and C; see Figure 3D. This node was modeled in a triplet spin state. This structure has an O–H BDFE of 53.8 kcal mol^–1^. Thus, all three possible proton topologies of reduced node with missing-linker defect sites have BDFEs lower than the experimentally measured values.

As described further in the Discussion section, we believe these calculations indicate that missing-linker defect sites are either largely absent in all of the crystallites or have a minimal role in the HAT reactions probed by *E*_OCP_ measurements.

## Discussion

### Proton-to-Electron Stoichiometry in the Redox Reaction of Ti-MIL-125 with Various Crystal Sizes

Three different crystal sizes of Ti-MIL-125, denoted **Ti-MIL-125-S**, **-M**, and **-L**, each containing different amounts and possibly different types of structural disorders, were employed to examine their effects on charge transfer thermodynamics. These include proton-coupled electron transfer (PCET) reactions and individual proton/electron transfer (PT/ET) reactions.

In the PCET reactions of Ti-MIL-125, the proton-to-electron stoichiometry remained as 1H^+^/1e^–^, regardless of the crystal size. A defect-free Ti_8_ node contains eight μ_2_–O, which can be protonated during a PCET reaction.^28–30^ The so-called missing-linker defect sites will further introduce more bases per node.^42–44^ Given the high density of Brønsted base within one node, it is surprising that all Ti-MIL-125 crystallites exclusively undergo 1H^+^/1e^–^ redox reaction.

Charge compensation is often used as the rationale for equimolar stoichiometry of protons and electrons in a PCET reaction.^16^ When, for example, more protons than electrons are added per redox-active site, the system will be net positively charged and therefore can be unstable. In fact, charge compensation is the basis of energy-storage devices where cations like Li^+^ within Li-ion batteries are intercalated into the electrode with equimolar amounts of electrons.^53–55^

However, many metal oxides can exhibit PCET stoichiometry beyond a 1:1 ratio, particularly when they are porous.^56^ An example of this is the comparison of hydrous vs anhydrous IrO_*x*_. When IrO_*x*_ is hydrated, with surface-bound and intralattice H_2_O molecules, it exhibits a Pourbaix slope between 90–120 mV pH^–1^, suggesting 3H^+^/2e^–^ to 2H^+^/1e^–^ stoichiometry.^57–59^ The extra positive charge(s) are thought to be compensated through anions within the electrolyte.^60^ Instead, when IrO_*x*_ is dehydrated, the system exhibits the Nernstian slope of ∼59 mV pH^–1^.^61,62^ Layered double hydroxides (LDHs),^63,64^ and more recently specific trap state of TiO_2_,^22^ can undergo PCET reactions with up to 2H^+^/1e^–^ stoichiometry. This so-called super-Nernstian behavior may occur within redox-active MOFs, which exhibit even higher porosity than LDHs or hydrous oxides. Yet, regardless of the buffer or the pH, all crystallite sizes of Ti-MIL-125 exhibited 1H^+^/1e^–^ stoichiometry.

The strict adherence to 1H^+^/1e^–^ stoichiometry during the PCET reaction of Ti-MIL-125, regardless of their crystal sizes, suggests two interesting phenomena. First, even though MOFs may be “more similar than different” to LDHs or hydrous metal oxides with pores filled with H_2_O/ions, the Ti-MIL-125 nodes strongly prefer to retain charge neutrality. Furthermore, any structural disorders within Ti-MIL-125 do not behave like some trap states of TiO_2_, which prefer a 2H^+^/1e^–^ PCET reaction. If Ti-MIL-125 undergoes a 2H^+^/1e^–^ PCET reaction, the nodes are net-positively charged and therefore must be charge-compensated by anions; borate anions with a small kinetic diameter can diffuse throughout the lattice, while sterically more demanding MES cannot.^27,28^ Tris or its protonated form, [TrisH]^+^, cannot participate in this charge compensation. Yet, as shown in Figure 4, *E*° of each crystallite in various buffers referenced against RHE are *similar to each other*. This strongly supports the 1H^+^/1e^–^ stoichiometry during the PCET reaction of Ti-MIL-125. When proton-to-electron stoichiometry is identical, both the oxidized and reduced nodes are overall charge-neutral. E­(ζ) values for all Ti-MIL-125 crystallites in MES/borate buffers were overall negative, suggesting that these anions are *not* participating in any charge compensation. We note that *E*° values are different between different crystallites, and we elaborate on this in the next section.

**Figure 4:**
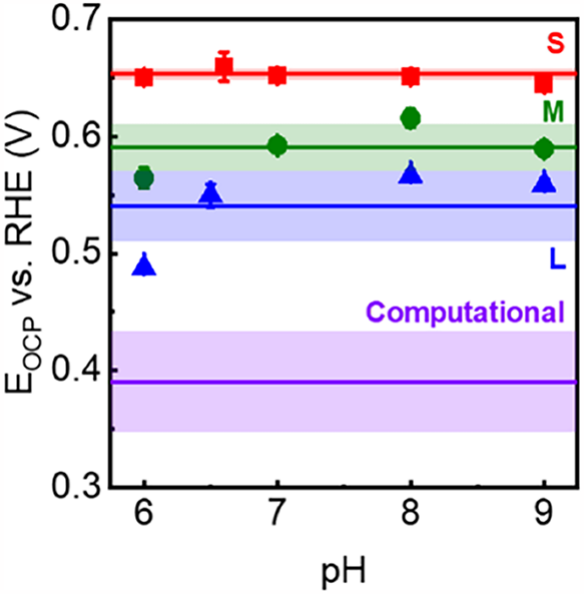
*E*° vs RHE of Ti^4+^O/Ti^3+^O–H redox reaction at Ti_8_ nodes of **Ti-MIL-125-S**, **-M**, and **-L**. These values were derived from *E*° vs NHE of the same redox reaction plotted in Figure 2D. The horizontal lines and the surrounding shaded regions indicate the average and 1σ values of each crystallite, respectively. The average and 1σ of computational values of the same redox reaction derived in our previous publication^30^ are also shown.

In sum, the proton-to-electron stoichiometry of Ti-MIL-125 remains one-to-one regardless of the crystal size, buffer, and pH of the electrolyte. With the stoichiometry established, we discuss their implications on the thermodynamics of PT, ET, and PCET reactions in the next sections.

### Crystal-Size-Dependent Thermodynamic Potential of Ti^4+^O/Ti^3+^O–H Redox Couple

The above establishes that the crystal sizes of Ti-MIL-125 have a minimal role in the PCET stoichiometry probed using *E*_OCP_ measurements. *However, the electrochemically derived PCET thermodynamics of Ti-MIL-125 were highly dependent on their crystal sizes.*

Surface charges are often considered to play a critical role in PCET reactions.^65,66^ The fully deprotonated surface is enriched with negative charges, which should repel electrons and therefore facilitate oxidation. Similarly, a positively charged surface due to moieties like [M–(OH_2_)]^+^ will instead promote reduction. We can rule out that charges play little to no role in the PCET mechanism of Ti-MIL-125 of all crystallites, as *E*_OCP_ and *E*(ζ) have very distinct trends versus the pH of the employed electrolytes. Thus, it is more appropriate to consider the PCET reaction of Ti-MIL-125, regardless of the crystal sizes or electrolyte, as a homolytic bond formation/cleavage of the Ti^3+^O–H/Ti^4+^O couple (like that shown in Scheme 2). We direct the readers to our previous publication for more details.^30^

The *E*° values of Ti^4+^O/Ti^3+^O–H redox reaction at Ti_8_ nodes referenced against RHE are thermochemically equivalent to the free energy of hydrogenation (Scheme 2). The free energy of this reaction is different from Ti^3+^O–H BDFE solely by a constant, Δ*G*°_f_(H•). Here, we emphasize that the ± 2 kcal mol^–1^ errors common for many BDFE values are largely due to the error on Δ*G*°_f_(H•).^9^ Thus, by directly comparing *E*° vs RHE, the associated errors in HAT thermodynamics are greatly reduced. As shown in Table 2, without the error of Δ*G*°_f_(H•), the difference in *E*°(Ti^4+^O/Ti^3+^O–H) between different crystallites is more prominent. While we acknowledge that the difference in *E*°(Ti^4+^O/Ti^3+^O–H) between **Ti-MIL-125-M** and **-L** is small (and we explain our rationale behind this later), it is still tempting to claim that crystal size has an impact on the thermodynamics of the HAT reaction at Ti_8_ nodes of Ti-MIL-125.

Figure 4 further illustrates the apparent cathodic shift in *E*°(Ti^4+^O/Ti^3+^O–H) with the increase in crystal size. In the same figure, we have also plotted the computed *E*°(Ti^4+^O/Ti^3+^O–H) of a pristine node (with no missing-linker defect sites) from our previous work.^30^ Ideally, this is the expected BDFE of a defect-free Ti-MIL-125 that is infinitely large in crystal size. This value was derived from the computed Ti^3+^O–H BDE using eq 6; entropic components were omitted because of the arbitrarily high translational entropy of H• prior to its binding to the node. It is possible that the difference between computed vs experimental *E*°(Ti^4+^O/Ti^3+^O–H) may altogether be due to this lack of entropic components. However, for molecular species, entropic contributions are usually larger than the differences observed here.^9^ Thus, we believe this difference indicates that structural disorder induced by the change in crystal size plays a prominent role in defining the HAT thermodynamics of Ti-MIL-125. If this is true, Figure 4 shows that H atoms on larger crystals are stronger reductants than those on the smaller crystals.

In this scheme, VB and CB represent valence and conduction bands, respectively. The proton-to-electron stoichiometry of TiO_2_ trap states can range between 1:1 to 2:1. This is why in Scheme 3A, m and n are used to denote these stoichiometries. The exact BDFE of TiO_2_ CB is, to the best of our understanding, unknown, so it is not specified in Scheme 3A. The BDFE distribution of each energetic state is indicated by the width of the rectangles. In Scheme 3B, two of many computational structures representing defect-free vs missing-linker defect sites are shown. The structure for a defect-free node was derived in our previous publication.^30^

To the best of our understanding, *this is the first report quantifying the effect of structural disorder on the HAT thermodynamics of heterogeneous materials.* In the next section, we discuss chemistry that may be behind this difference in thermodynamics.

### Missing-Linker Defect Sites vs Structural Distortion of Ti Cations and Their Effects on Ti^3+^O–H BDFE

We have previously demonstrated that the computationally predicted Ti^3+^O–H BDFE values of a pristine node range from 61 to 63 kcal mol^–1^, regardless of the number of H atoms or the proton topology.^30^ In this work, we elaborated on this by computing the Ti^3+^O–H BDEs at the missing-linker defect sites. All simulations indicate that electrons are localized on the Ti^3+^ cation while the proton is bound to the nearby Brønsted base (see Figure 3 and ref 30). Localization of electrons has also been observed in the simulations reported by Fabrizio et al.^29^ BDEs at the missing linker defect sites are lower by 4–10 kcal mol^–1^ than those on pristine nodes, and any of the experimentally derived values. As shown in Figure 4, an *increase* in the crystal size leads to *a cathodic shift* in *E*_OCP_ vs RHE, corresponding to a *decrease in Ti* ^*3+*^ *O–H BDFE*. We have previously demonstrated that a decrease in crystal size induces more structural disorder at the surfaces of Ti-MIL-125.^31^ Thus, **Ti-MIL-125-S** with the highest amount of surface Ti sites should, if at all, have the highest amount of disorders. Similar concepts have been invoked for Cu, Zn, and Zr-based MOFs.^67–70^ Yet, Ti^3+^O–H BDFE of **Ti-MIL-125-S** is higher than those of **Ti-MIL-125-M** or **-L**. Therefore, we claim that the primary reason for the difference in PCET thermodynamics between different crystal sizes of Ti-MIL-125 is due to the *local geometric distortion of Ti cations, not due to the presence of missing-linker defect sites.* We note that local geometric distortion of Ti-MIL-125 has recently been observed through high-resolution, integrated differential phase contrast STEM images by Feng et al.^71^ Though the crystals were >500 nm in size, it is conceivable that similar distortions exist within the colloidal Ti-MIL-125 employed in this study.

Missing linker defect sites can be identified from pore size distribution analysis; N_2_-adsorption–desorption isotherms of defective Ti-MIL-125 typically exhibit a mesopore with an N_2_ uptake at P/P_0_ of ∼0.3.^42–44^ For all employed Ti-MIL-125 crystallites in this study, this was not observed. These mesopores are typically introduced only when Ti-MIL-125 crystals are exposed to “harsh” conditions, including, but not limited to, O_2_ plasma treatment or exposure to strong bases/acids. Thus, we conclude that “missing-linker” defect sites are likely playing no major role in the PCET reactions of Ti-MIL-125 crystallites presented in this study.

### Comparisons of Disorder-Dependent Ti^3+^O–H BDFE of Ti-MIL-125 to TiO_2_ and Molecular H Atom Donors/Acceptors

We start this section by emphasizing that for all crystal sizes of Ti-MIL-125, their O–H BDFEs are higher by ca. 20 kcal mol^–1^ than those of TiO_2_. Typically, Ti^3+^O–H BDFE of TiO_2_ ranges between 39–49 kcal mol^–1^.^9,72,73^ We have previously ascribed this large difference in BDFE between Ti-MIL-125 and TiO_2_ to be due to the unique ring-like node structure of Ti-MIL-125.^30^ The results presented here further corroborate this; local geometric distortion of Ti cations within the node retains the ring-like structure.

The band energy diagrams of TiO_2_ and Ti-MIL-125 with structural disorders like trap states further highlight the distinct PCET chemistry between the two systems. A typical band energy diagram of TiO_2_ with a trap state is reproduced in Scheme 3A;^10–12^ here, we prefer to report all energies as BDFEs, which are independent of the proton activity of the electrolyte.^9^ The proton-to-electron stoichiometry is deliberately kept arbitrary using *m* and *n* in Scheme 3A as it can vary (*vide supra*). In Scheme 3B, we show a similar energy diagram of Ti-MIL-125. The *E*_g_ from the valence band (VB) is from our measurements (see Table 2). The BDFE of the conduction band (CB) is equivalent to those measured experimentally for Ti-MIL-125 of all crystallites, agreeing with those reported by Hendon et al.^6^ The “trap state” that is structurally distinct from CB is essentially the missing-linker defect sites that we have computationally demonstrated. As noted above, these sites have lower BDFE. H atom migration from CB to these trap states in Ti-MIL-125 is endergonic, and as described above, they are likely not present in any of the crystallites examined in this study. Notably, PCET reaction of TiO_2_ is thought to almost exclusively occur at the trap states, with H^+^/e^–^ stoichiometry ranging between 1:1 to 2:1.^14,15^ In our presented study, however, based on our computational calculations, E_OCP_ measurements are likely probing the thermodynamics of what would otherwise be considered CB energy, which is the active site for PCET reaction.

**Scheme 3:**
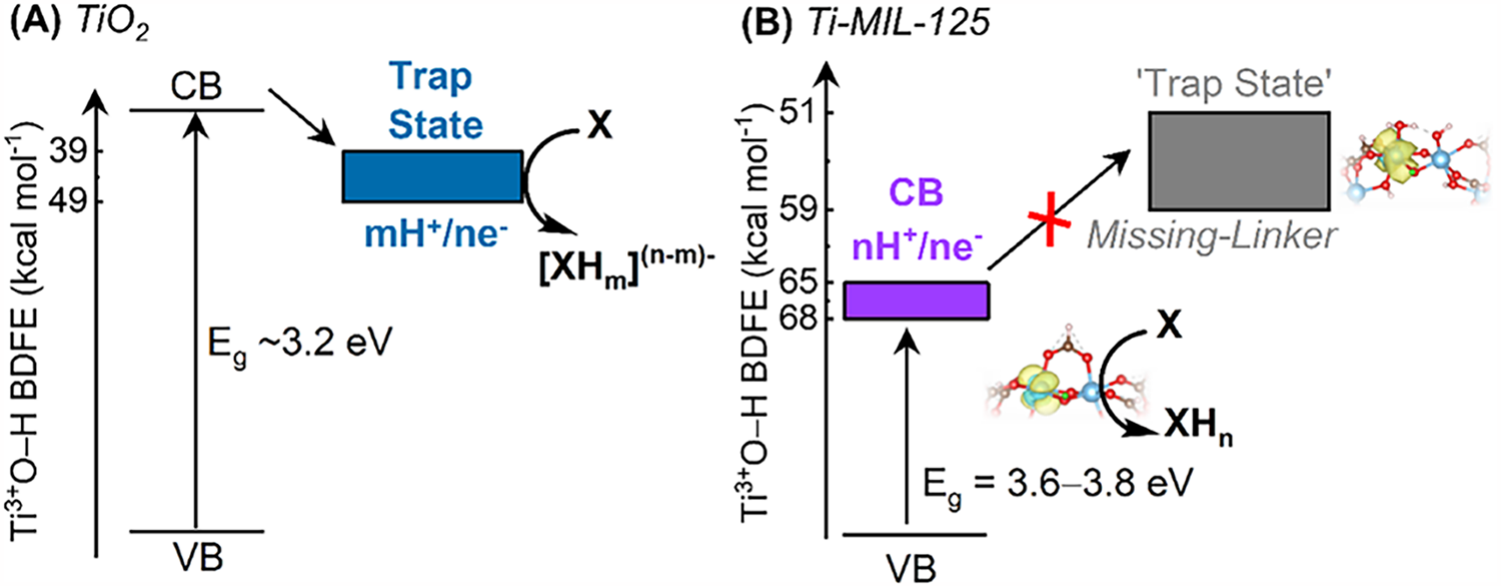
Band Energy Diagrams of (A) TiO_2_ and (B) Ti-MIL-125. Reactions with a Generic Substrate, X, are used to Emphasize the Precise Moiety at Which the PCET Reaction Occurs. The width of the CB/trap state energies indicate their distributions.

The ∼3 kcal mol^–1^ range in BDFE between nodes of different crystal sizes (or 6 kcal mol^–1^ if to include the *in-silico* model) is similar to those of molecular H atom donors and acceptors with identical backbone but with different functional groups. The average O–H BDFE of 1,4-hydroquinone with various functional groups, for example, can vary between 62 to 69 kcal mol^–1^. Similar differences are observed between variants of 2,2,6,6-tetramethylpiperidine (TEMPO–H), phenols, and anilines.^9^ Molecules with various functional groups are treated as *different molecules* with unique oxidizing/reducing strength (i.e., driving force to donate or accept H atoms). Similarly, here, we advocate that *Ti-MIL-125 of various crystal sizes should be treated as different chemical entities with distinct reactivity in HAT reactions.*

### PT/ET Reactions and Their Thermodynamics of Ti-MIL-125

Much like the PCET thermodynamics, the thermodynamics of PT and ET were also highly dependent on the crystal sizes of Ti-MIL-125.

The UV–vis spectra of **Ti-MIL-125-S**, **-M**, and **-L** were examined previously to demonstrate that the optical properties of the Ti^3+^ cation are heavily dependent on the crystal size.^31^ This parallels the notion of geometric distortion of Ti cations, mentioned above, that inevitably changes the *d*-to-*d* transition energies.

Furthermore, UV–vis spectra of **Ti-MIL-125-S**, **-M**, and **-L** in their pristine forms were used to determine *E*_g_ values. As described above and by Hendon and coworkers, this *E*_g_ probes the linker-to-metal charge transfer reaction;^6^ all values are well-above the energy of that of visible light, which agrees with the need for UV irradiation in the presence of a sacrificial reductant for the photoreduction. *E*_g_ values systematically shifted to a lower value as the crystal size increases (Table 2 and Figure 2A), approaching the computed value of 3.6 eV.^29^ This further corroborates the notion of geometrically distorted sites being more prevalent as the crystal size decreases.

In addition to ET thermodynamics, our potentiometric titrations suggest that PT thermodynamics is also highly sensitive to the crystal sizes of Ti-MIL-125. Mian et al. have previously reported the p*K*_*a*_ of the Ti–OH–Ti moiety within the node to be ca. 4.9.^35^ This *does not* match any of the p*K*_*a*_ values found in this study. For all crystallites, we observed two p*K*_*a*_ values; the second value, denoted p*K*_*a*,*B*_ in Table 2, is ascribed to the same chemical moiety as Mian et al. (denoted H_B_ in Figure 2B). The ca. 10 times increase in acidity is ascribed to the geometric distortion of the node that exists even for **Ti-MIL-125-L**. The other p*K*_*a*_ value (p*K*_*a,A*_) is significantly low, where the relevant Brønsted acid is at least 1000 times more acidic than the Ti–OH–Ti moiety mentioned above. We ascribe this to the protonation of one or more of the eight μ_2_–O that bridges between two Ti cations (denoted H_A_ in Figure 2B); these are typically drawn deprotonated, like that shown in Figure 1, to keep the node charge-neutral. Among the three crystallites, the p*K*_*a*_ values of **Ti-MIL-125-S** were statistically distinct from those of the other two crystallites, **Ti-MIL-125-M** and **-L**. Given the crystal size, it is likely that **Ti-MIL-125-S** is rich in Ti_8_ nodes that are geometrically distorted, and the Ti–O bonds within each node deviate the most from those of the ideal node.

The above p*K*_*a*_ measurements suggest that the nodes of Ti-MIL-125 are fully deprotonated within the pH range employed for all electrochemical measurements (pH = 6–9). This agrees with the E­(ζ) measurements, where in MES and borate buffers, regardless of the crystal sizes, the values were negative. *E*(ζ) was nearly zero in a pH 7-adjusted Tris buffer. At such a pH, Tris molecules are largely protonated and thus can compensate for the negative charges on the nodes.

The observed strong dependence of both p*K*_*a*_ and electron energetics on the *exact chemical nature* of active sites parallels many other findings using TiO_2_ and other heterogeneous materials. The Pourbaix diagram of TiO_2_ reported by Lyon and Hupp suggests that the surface O–H groups protonate and deprotonate only at extreme conditions where the Hammett acidity parameters (H_0_) of the surrounding solutions were <0 and >20, respectively.^74^ Other computational and experimental results have pointed toward a complex dependence of the p*K*_*a*_(TiO–H) on the lattice structure, orientation of water molecules at the solid–liquid interfaces, and charges of the surrounding species.^75–78^ These factors can shift the p*K*_*a*_ of TiO_2_ sometimes by seven p*K*_*a*_ units; this corresponds to ∼10 kcal mol^–1^ difference in proton transfer energy at 298 K. Similarly, at a given pH, the band gaps of nominally identical “TiO_2_” can differ by ∼0.5 V or 12 kcal mol^–1^.^79^ Differences in lattice structure due to dopants, impurities, and interfacial interactions with ions and solvents all lead to drastic differences in the PT/ET energetics.

The square scheme shown below summarizes the relevant ET/PT/HAT reactions of Ti-MIL-125 discussed in this study (Scheme 4). The ET/PT reactions yield charged, and therefore likely unstable, products. At pH 6–9, nearly all of the relevant μ_2_–O are protonated, given that the p*K*_*a*_ is ≤1.9. E_g_ values of ≥3.7 eV suggest that under *E*_OCP_ measurements, pristine Ti-MIL-125 cannot undergo ET reaction. This agrees with the previous report by Saouma et al., where a minimal amount of reduction was observed when Ti-MIL-125 was exposed to strong reductants like decamethylchromocene in the absence of protons or other counter cations.^80^

**Scheme 4:**
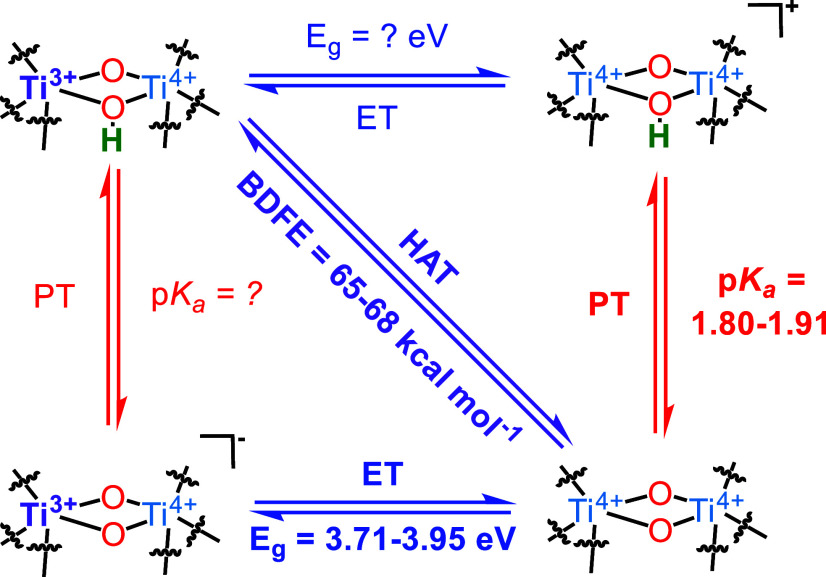
Square Scheme Illustrating PT/ET/HAT Reactions of Ti-MIL-125

The p*K*_*a*_ and band gap of charged species at the top right-hand and bottom left-hand corners were not determined due to their instability under *E*_OCP_ reaction conditions. Only one out of many possible proton topologies upon photo reduction is shown above. Other proton topologies had similar BDFE as long as only one node was considered. See the following references^30^ and the next section for more details.

The square scheme deliberately shows the range of PT/ET/HAT thermodynamics of Ti-MIL-125, as these values depend on the crystal size. These highlight the importance of considering structural disorders in the thermodynamics of these reactions. These parameters often dictate the reactivity of MOFs, but the effect of subtle defects like the geometric distortion of nodes on catalytically relevant thermodynamics is often overlooked. The exact correlation between PT/ET/HAT energetics and defect sites remains rare in the MOF literature.

### Implications of Size-Dependent Ti^3+^O–H BDFE on “Bulk-to-Surface” H Atom Migration of Ti-MIL-125

As noted above, Ti^3+^O–H BDFE increases as the crystal size decreases. By assuming crystallites to be a perfect sphere, and by using the crystallographic parameters of Ti-MIL-125 reported previously,^27^ we can determine that >50% of Ti_8_ nodes within **Ti-MIL-125-S** are exposed at the surface. This value decreases to ∼30% for **Ti-MIL-125-L**. For further details on the calculation of surface vs bulk nodes, see ref 31 These calculations suggest that the *E*_OCP_ values of **Ti-MIL-125-S** must be more sensitive to the PCET reactions at the nodes within the surface of the crystallites.

The experimentally determined difference in *E*° vs RHE/ΔBDFE between **Ti-MIL-125-S**, **-M**, and **-L** suggests that the Ti_8_ nodes at the crystal surface have a larger driving force to accept H atoms than those within the bulk. *E*_OCP_ measurements probe the average of all H atoms; thus, none of the reported values solely probe Ti^3+^O–H BDFE of surface sites. Still, ΔBDFE of −2.5(7) kcal mol^–1^ between **Ti-MIL-125-S** and **-L** suggests that under identical reaction conditions, H atoms are more likely to be at the surface than in bulk, at least by a factor of 60–70 (eq 2).^81^

8 $$\Delta BDFE=-RT\ln\left(K_{eq}\right)$$

This can explain the so-called H atom migration that has been previously observed for Ti-MIL-125.^28^ When reduced Ti^3+^-MIL-125 was exposed to an H atom acceptor like 2,4,6-tri*t*-butylphenoxyl radical (2,4,6-*t*-Bu-PhO^•^), all Ti_8_ nodes were oxidized, forming 2,4,6-tri*t*-butylphenol (2,4,6-*t*-Bu-PhOH). While this HAT should be exergonic as the O–H BDFE of 2,4,6-*t*-Bu-PhOH is 75 kcal mol^–1^, the complete oxidation of the MOF is surprising as the phenoxyl radical is too sterically demanding to diffuse through the MOF pores. The complete reaction suggests that H atoms “hop” from the bulk of the crystals to the surface, but the exact reason for such behavior was unknown. Here, based on our electrochemical/computational measurements, we claim that this hopping is, at least in part, driven thermodynamically. Based on the ΔBDFE values, there is a driving force of at least 3–6 kcal mol^–1^ for the nodes within the bulk to donate H atoms to those on the surface. This also agrees with the similar titration studies mentioned above, but using a much weaker H atom acceptor, TEMPO^•^. Given its size, TEMPO^•^ can readily diffuse into the pores of the MOF. Thus, even though TEMPO–H has a weaker O–H BDFE of 66 kcal mol^–1^, the nodes within the bulk with Ti^3+^O–H BDFE of ≤65 kcal mol^–1^ can still donate H atoms.

### Non-Langmuirian H Atom Adsorption–Desorption Isotherms of Ti-MIL-125

We and others have demonstrated that each node of Ti-MIL-125 can accept up to two H atoms, or 2H^+^/2e^–^, and when defect-free, their Ti^3+^O–H BDFE values are nearly identical.^28–30^ Thus, we have previously concluded that H atom adsorption/desorption thermodynamics on Ti-MIL-125 must follow the *ideal* Langmuir isotherm. While this is true within one node, the presented work suggests that when H atoms within the entire crystallite are considered, the *Langmuir isotherm is no longer an accurate model*.

Deviation from the Langmuir model is often associated with lateral interactions between adsorbates and/or chemical heterogeneity.^82–87^ For Ti-MIL-125, our computational simulations suggest that linkers are minimally distorted; we have also demonstrated previously that the PXRD patterns of reduced Ti-MIL-125 are identical to those of the pristine form.^30^ Thus, at least an extensive lattice distortion is unlikely in Ti-MIL-125. Each node within the lattice is separated by ∼7–10 Å based on the crystal structure,^27^ and hence, we expect the nodes not to undergo a “through-space” lateral interaction. As noted above, two H atoms within a single node are very similar in their BDFE values. *Thus, the observed non-Langmuirian behavior of H atoms on Ti-MIL-125 can be ascribed primarily to the chemical heterogeneity.* ^88^

We emphasize that the two most common reasons for non-Langmuirian isotherms, lateral interaction vs chemical heterogeneity, are hardly ever distinguishable.^38,89^ Underpotential deposited hydrogen (H_UPD_) on a single-crystalline Pt(111) surface is one of the few rare cases where lateral interactions between the H_UPD_ are ascribed to be the primary reason for the *nonideality*.^90^ Even in this case, the choice of electrolytes and pHs is limited to prevent the adsorption of species other than H atoms.^91^ For binary materials with complex and dynamic interfacial structures, it is nearly impossible to decipher whether one or more factors play a role in H atom adsorption/desorption.

We also reported the difficulty in distinguishing between these two phenomena in Ce-MOF-808.^50^ H atom adsorption on hexanuclear Ce-oxo nodes of Ce-MOF-808 induces lattice strain due to the expansion of Ce cations by ∼50% in volume. Ce-MOF-808 also presents multiple proton topologies with distinct BDFEs.

In the presented work with Ti-MIL-125, over a wide range of pH and electrolyte, we can conclude that the BDFE distribution is most likely due to the distinct HAT chemistry at the surface vs the bulk of Ti-MIL-125 crystallites. The nodes at these two sites are, essentially, distinct chemical species.

### Emphasis on Non-Ideality of Ti-MIL-125 in HAT Reactions

Here, we want to emphasize that much like other binary materials, the PT/ET/HAT thermodynamics of Ti-MIL-125 are highly dependent on the exact chemical nature of the active sites.

Chemistry at defect sites of MOFs has been invoked for decades.^92^ Missing-linker or missing-node defect sites can increase the apparent pore aperture to facilitate substrate/product diffusion and access to catalytically active sites. Klet et al. have further quantified thermodynamic parameters like p*K*_*a*_ values of Brønsted acids at defect sites of UiO-series.^32^ In all cases, because of the well-known crystal structure of the MOFs, the invoked defect site structures are more likely to be structurally representative of the actual system. This contrasts with those within metal oxides, where the catalytic sites are often amorphous and therefore structurally ambiguous. This is precisely how we and others have invoked missing-linker defect sites in Ti-MIL-125, and in this case, conclude that these defect sites are irrelevant to the presented studies. Indeed, recent works using high-resolution electron microscopy images have structurally identified missing-linker and missing-node defect sites within MOFs like UiO-66 or MIL-101, either within the bulk or at the surface of crystallites.^93,94^

Our findings also emphasize that the catalytically relevant thermodynamics of MOFs like Ti-MIL-125 are *highly dependent* on the exact system. This challenges an implicit assumption common within the field of MOF-based catalysis. Often, MOFs are considered as a “single site” catalyst where structurally uniform catalytic motifs are presented within the system. This, therefore, suggests a single value for a catalytically relevant thermodynamic parameter for the reactions occurring throughout the crystals.^49,95–98^ This is certainly not a valid assumption for PT/ET/HAT reaction in Ti-MIL-125, as the thermodynamic parameters of these reactions are *crystal size dependent.* It is likely that this assumption is not valid for many other MOF-based catalysis.

### Limitations of the Presented Studies

We conclude the Discussion section by describing the limitations of this study. Limitations specific to *E*_OCP_, band gap, and p*K*_*a*_ measurements can be found in the following references.^30,32,99^

As shown in Scheme 5, the particle size distributions of **Ti-MIL-125-S, -M,** and **-L** all have significant overlaps. Notably, an increase in crystal size often leads to a broader distribution range.^29^ Thus, some particles within a batch of **Ti-MIL-125-S**, for example, may have BDFEs like those within **Ti-MIL-125-L**. This is likely why the difference in BDFEs of **Ti-MIL-125-M** and **-L** was small (see the overlap in Scheme 5). Similar arguments can be made for *E*_g_, *d*-to-*d* transition energies, and p*K*_*a*_ values. All measurements presented in this work probe the *average* values of the entire suspension.

**Scheme 5:**
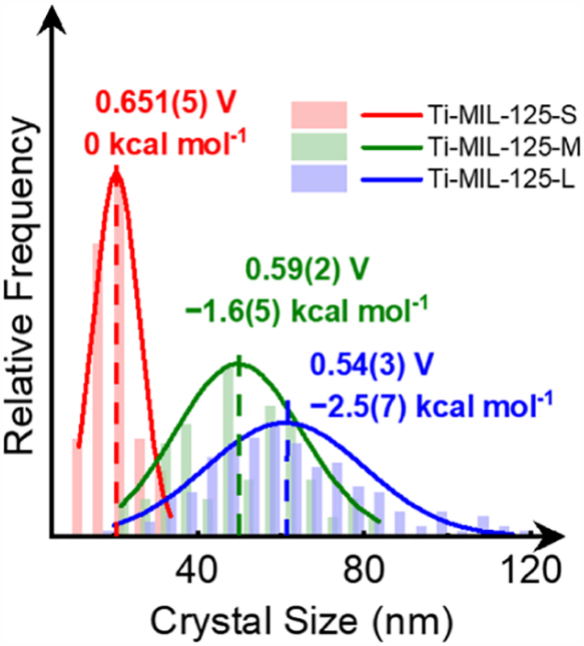
Schematic Illustration Showing Size Distribution of Ti-MIL-125-S, -M, and -L and their *E*° vs RHE in V and ΔBDFE in kcal mol^–1^

At least for the BDFE, the width of the distribution is theoretically measurable using electrochemical techniques like cyclic voltammetry (CV). The fwhm of the Faradaic features directly correlates with the BDFE distribution.^36,38,50^ We have previously shown, however, that CVs of Ti-MIL-125 only exhibit cathodic features, and no anodic features. This precludes any thermochemical analysis.^30^ This limitation parallels those of *E*_OCP_-derived BDFEs of many molecular systems, outlined by Wise et al.^40^ For molecules like hydroquinone, the *E*_OCP_ related to the hydroquinone/semiquinone or semiquinone/benzoquinone couple cannot be measured. We emphasize, however, that for molecular species and binary materials (*e.g.,* NiO^100^ with multiple reactive H atoms, the average BDFE values are often an accurate proxy to determine their reactivity in HAT reactions.

Up to this point, we have deliberately used the words “structural disorder” and “local geometric distortion” to encompass a wide range of possible structural changes (*e.g.,* change in bond angle, distance, and symmetry). Attempts to probe these structural changes through spectroscopic techniques were unsuccessful, likely because the structural disorders were localized at the surface. High-resolution TEM images were also attempted, but it was difficult to identify any obvious structural disorders (see the Supporting Information).

Because of the above experimental difficulty, we turned to computational simulations to probe the structural disorders. This, unfortunately, also turned out to be difficult. There remains a question on how to computationally model the possible disorders systematically. Furthermore, the density functional theory (DFT) calculations employed in this study will be prohibitively expensive to probe ≥15 nm crystals, while the semiempirical quantum chemistry calculation is unlikely to probe subtle differences in BDFE observed here. Still, by computing BDFEs at other potential sites, we were able to conclude that geometric distortion is likely the reason for the difference in BDFEs.

Exploration beyond the reported crystal sizes also proved difficult. Synthesis of a crystal size below that of **Ti-MIL-125-S** resulted in poor crystal quality. This is expected as the unit cell of Ti-MIL-125 is roughly 1 × 1 × 1 nm.^27^ Long-term colloidal stability of crystals larger than **Ti-MIL-125-L** precluded accurate *E*_OCP_ measurements. Even for **Ti-MIL-125-L**, in a pH 7-adjusted Tris buffer, which induces near-zero surface charges, crystals agglomerated near-instantaneously; this is likely due to the lack of intercrystal electrostatic repulsion (see the SI). The Pourbaix diagrams and BDFEs of **Ti-MIL-125-M** and **-L** had larger 1σ than those of **Ti-MIL-125-S**; see Figures 2D and 4.

Despite the above limitations and challenges, our studies have *quantitatively* demonstrated the effect of defect sites on ET, PT, and HAT thermodynamics relevant to catalysis and beyond.

## Conclusions and Future Outlook

PCET reactions at Ti_8_ nodes of Ti-MIL-125 crystallites of three different crystal sizes were probed using *E*_OCP_ measurements; these findings have suggested that Ti-MIL-125, regardless of the crystal size, undergoes a 1H^+^/1e^–^ redox reaction. However, the thermodynamics of this PCET reaction, namely the Ti^3+^O–H BDFE, are highly dependent on the crystal sizes. N_2_-adsorption–desorption isotherms and computational calculations suggest that this difference in BDFEs is *not* due to the presence of missing-linker defect sites commonly proposed in Ti-MIL-125. Instead, the difference likely arises from small but significant geometric distortion local to the Ti^3+^ cation that alters the PCET thermodynamics. UV–visible spectra and potentiometric acid–base titrations further demonstrated that the geometric distortion alters the *E*_g_, *d*-to-*d* transition, and p*K*_*a*_ values of the TiO–H groups, probing either ET or PT thermodynamics. Together, we have quantitatively demonstrated that these thermodynamic parameters are intrinsically correlated to the exact chemical nature of the active sites within Ti-MIL-125.

The three types of thermochemical values measured in this study are extensively employed as catalytically relevant “descriptors” in heterogeneous catalysis. For example, zeolites with a high density of Brønsted acids are the industrial-scale catalysts for reactions like alkane cracking and dehydrogenation.^101^ MOFs with Brønsted acid sites are also considered a viable candidate for many of these reactions.^102,103^ Band energies are often employed to describe photo/electrocatalytic properties of semiconductors and MOFs;^7,74,104^ recently, however, we and others have suggested that for PCET/HAT reactions, BDFEs are a more accurate descriptor as band energies solely probe energies of electrons, and not protons.^2,9,30,50^ Indeed, BDFEs and related thermochemical values are considered to be the relevant thermodynamic descriptors in reactions of H_2_, O_2_, CO_2_, N_2_, and many other energy-relevant reactions.^105–107^

The observed size-dependent *E*_g_, p*K*_*a*_, and Ti^3+^O–H BDFE values of Ti-MIL-125 crystallites call into question whether MOFs, particularly when nanosized, can be considered “single site” catalysts. The difference in chemistry between the surface vs bulk of MOFs, zeolites, and other porous materials has been raised for decades.^108,109^ And yet, MOF-embedded catalysts are often *implicitly* assumed to be *single site* in nature, with a *single* thermodynamic parameter. Here, we have quantitatively demonstrated that this assumption does not hold for colloidal Ti-MIL-125, and perhaps this may be true for other MOFs. *We advocate that Ti-MIL-125 of different crystal sizes should be treated like chemically distinct H atom donors/acceptors, much like molecular species with different backbone/functional groups are considered distinct species.* ^9^

While the focus of this project was on transfer of H atoms, charge-transfer reactions involving O/N/S atoms also are relevant to energy, chemical, and biological sectors.^110–114^ Our current research focuses on examining the thermodynamics of all of these atoms and assessing the role of defect sites within MOFs. Together, these studies should comprehensively determine the exact role of defect sites and other structural disorders within MOFs in defining catalytically relevant thermodynamics and their reactivity.

## Supplementary Material



## Notes

Characterization and electrochemical results of all Ti-MIL-125 crystallites along with details on computational simulations (PDF)
