# Supplementary material for: Proton, Electron, and Hydrogen-Atom Transfer Thermodynamics of the Metal–Organic Framework, Ti-MIL-125, Are Intrinsically Correlated to the Structural Disorder
Source: J Am Chem Soc. 2025 Sep 13;147(38):34777–90. doi: 10.1021/jacs.5c10498 (PMC12464971; doi:10.1021/jacs.5c10498)
Supplement: Supplementary file 1 [file ja5c10498_si_001.pdf]

Supporting Information

Proton, Electron, and Hydrogen-Atom Transfer Thermodynamics of the Metal–Organic Framework, Ti-MIL-125, are Intrinsically Correlated to the Structural Disorder

Nazmiye Gökçe Altınçekiç,[†] *Chance W. Lander,*[†] *Jiaqi Yu,*[‡] *Ayman Roslend,*[‡] *Yihan Shao,*[†] *and Hyunho Noh*[†]*

[†]Department of Chemistry and Biochemistry, University of Oklahoma, Norman, OK 73019, USA

[‡]Department of Chemistry, Northwestern University, Evanston. IL 60208, USA

*Corresponding author

Hyunho Noh: hyunho.noh-1@ou.edu

Table of Contents

1 General Considerations ... 3
1.1 Materials ... 3
1.2 Instrumentation ... 3
2 Experimental Details ... 4
2.1 Details on open-circuit potential (E_{OCP}) measurements ... 4
2.2 Details on UV-Visible Spectra Measurements ... 5
2.3 Details on potentiometric acid-base titrations ... 5
2.4 Dynamic Light Scattering ... 5
3 Physical Characterization of Ti-MIL-125-S, -M, and -L ... 6
3.1 N_2 Adsorption-Desorption Isotherms for three Ti-MIL-125 crystallite batches ... 6
3.2 PXRD Patterns of Ti-MIL-125 and Scherrer Equation ... 7
3.3 STEM Images ... 7
3.4 TEM Images ... 9
3.5 DLS ... 9
3.6 UV-Visible Spectra ... 11
3.7 ^{1}H NMR Spectra ... 12
3.8 Zeta-Potential Measurement ... 12
4 Open-Circuit Potentials of Ti-MIL-125-M and -L ... 13
4.1 Results for Ti-MIL-125-M ... 14
4.2 Results for Ti-MIL-125-L ... 16
5 Details on the Computational Calculations ... 17
5.1 Ti_8 Nodes with H-atoms and their calculated Ti^{3+}O–H BDE/Bond Distance Changes 17
5.2 XYZ Coordinates of Computational Models ... 21
6 References ... 25

1 General Considerations

1.1 Materials

All chemicals and materials in this work were used as received.

The synthesis of Ti-MIL-125 employed the following chemicals. Titanium (IV) isopropoxide ($Ti(iOPr)_4$; 97%), benzoic acid (≥99.5%), and 1,4-benzenedicarboxylic acid (H_2BDC; 98%) were purchased from Sigma-Aldrich. Anhydrous *N,N'*-dimethylformamide (99.8%), and methanol (≥99.8%) were acquired from Thermo Scientific.

The following chemicals were used to prepare pH-adjusted, aqueous electrolytes. Boric acid (H_3BO_3; ≥99.5%) and sodium chloride (≥99.0%) were supplied from Sigma-Aldrich. Tris(hydroxymethyl)aminomethane (Tris) was obtained from Fisher Scientific. 2-(N-morpholino)ethanesulfonic acid (MES; >99.0%) was acquired from TCL. For pH adjustments, concentrated hydrochloric acid (Fisher Scientific) and sodium hydroxide (Macron Chemicals) aqueous solutions at concentrations greater than 5 M were used.

Deuterated NMR solvents, D_2O (99.9 atom % D) and sodium deutroxide (40 wt% in D_2O, 99+ atom % D), were acquired from Sigma-Aldrich.

Ti-MIL-125 of three crystal sizes, referred to as **Ti-MIL-125-S**, **-M**, and **-L** in the main text and here onwards, were synthesized following the established procedure, using $Ti(iOPr)_4$ as the titanium source.[1] The resulting crystallites were suspended in DMF until further use.

1.2 Instrumentation

N_2-adsorption-desorption isotherms were measured using 3Flex (Micromeritics) to confirm the porosity of Ti-MIL-125 crystallites. Before conducting isotherm measurements, colloidal suspensions of Ti-MIL-125 were solvent-exchanged, and thermally activated under a dynamic vacuum (<50 mTorr) at 140 °C using VacPrep (Micromeritics), following the reported procedure.[2] The Brunauer –Emmett–Teller (BET) area of Ti-MIL-125 was calculated to be between 1200 – 1400 m^2/g based on the data set derived from the P/P_0 range of 0.005 – 0.1, which agrees well with other reports.[1,3] The isotherm and the DFT-derived pore size distribution for all the crystallites are reported below.

Ti-MIL-125 suspensions in methanol were photo-reduced using Rayonet photochemical reactor equipped with UV-A and UV-B light sources.

UV-visible spectra measurements of crystallites were conducted using a Cary 60 Spectrophotometer (Agilent). In all measurements, a custom-made 1 × 1 cm cuvette sealed with a septa was utilized to isolate the suspensions from ambient air. Optical band gaps acquired from Tauc plots are presented in *Section 3.5*.

The powder X-ray diffraction (PXRD) patterns of all crystallites were collected using the Rigaku Miniflex600 equipped with a Ni-filtered Cu Kα X-ray source, in the 2θ range of 3 to 90° and a step size of 0.02°.

Scanning transmission electron microscopy (STEM) and transmission electron microscopy (TEM) images of Ti-MIL-125 were obtained using the JEOL ARM200CF. Ti-MIL-125 crystallites were dried at 80 °C under vacuum, followed by methanol re-dispersion and drop-casting onto Cu grids, which were left to dry in air. Size estimation of the crystallites was performed using ImageJ.

Dynamic light scattering (DLS) measurements were performed using a Litesizer 500 instrument (Anton Paar GmbH), equipped with a 658 nm laser source. The instrument collected the intensity autocorrelation function $g_2(\tau)$ across various delay times, which were further analyzed using cumulant fitting to attain particle size distributions. The average of at least three measurements for all crystallite sizes is shown below.

For all crystallites of Ti-MIL-125, estimation of the concentrations were estimated using ^{1}H NMR spectra (Varian VNMRS 400 MHz). Approximately 1 M NaOD in D_2O was used to digest the MOFs to analyze the chemical shifts of linker-bound H-atoms. (vide infra for details).

Zeta potential measurements were performed with the Malvern Zetasizer Ultra. A 1:5 methanol-to-water mixture was prepared by adding Ti-MIL-125 suspension in methanol to aqueous buffers for determining the surface charge.

All open-circuit potential measurements were conducted using CH Instruments potentiostats (models 600D). Glassy carbon (GC) working electrode, which was obtained from CH Instruments, was polished with 0.5 μm alumina before all electrochemical measurements. The reference and counter electrodes, Ag/AgCl (3 M KCl) and Pt wire, respectively, were acquired from BASi. After all the measurements, potentials were referenced to the Normal Hydrogen Electrode (NHE).

The SevenDirect SD20 with the InLab® Expert Pro-ISM sensor from Mettler-Toledo was used for monitoring pH values of aqueous buffers and conducting potentiometric acid-base titrations. All pH measurements were taken after a standard four-point calibration, using buffers at pH 1.68 from Sigma Aldrich, and pH 4.01, 7.00, and 10.01, which were purchased from Oakton.

2 Experimental Details

2.1 Details on open-circuit potential (E_{OCP}) measurements

For all crystallite sizes, sample preparation and open-circuit potential (E_{OCP}) measurements were carried out as described in our previous report.[4] Unless otherwise stated, all measurements were performed using a standard Schlenk line with N_2 gas. All solutions were degassed through N_2 bubbling for at least 15 minutes to prevent oxidation of photo-reduced Ti^{3+}-MIL-125.

Briefly, a known volume of the colloidal suspension of Ti-MIL-125 in a custom-made cuvette with a septa seal was injected into the electrolyte. The suspension was then photo-reduced, which the duration depended on the crystal size (see the following references for details). This photo-reduced suspension was injected into the electrolyte while E_{OCP} was monitored. The photo-reduced suspension was titrated multiple times. These experiments were conducted using various buffers and pHs, as described in the main text. For each electrolyte, at least two measurements were performed to ensure reproducibility.

2.2 Details on UV-Visible Spectra Measurements

The UV-Visible spectra of Ti-MIL-125 samples prior to the photo-reduction, described in the following reference,[5] were used to estimate the band gap (E_g) using the Tauc plot. Concentrations of Ti-MIL-125 of all crystal sizes were limited to ~300 μM to minimize complications due to scattering. Representative UV-Visible spectra of **Ti-MIL-125-S**, **-M**, and **-L** are shown in *Section 3.5*.

E_g values of each crystallite were calculated using the Tauc equation shown below (eq. S1), following the reported procedure. In this equation, h, ν, and α represent the Planck's constant, frequency, and the absorption coefficient, respectively. Tauc plots of **Ti-MIL-125-S**, **-M**, and **-L** are shown in Figure 2A of the main text.

$$(\alpha h\nu)^2 = A(h\nu - E_g) \quad \text{(S1)}$$

2.3 Details on potentiometric acid-base titrations

Potentiometric titrations of Ti-MIL-125 followed the reported procedure.[6] Briefly, colloidal suspensions of **Ti-MIL-125-S**, **-M**, and **-L** were solvent exchanged from DMF to MeOH, then to aqueous solution of KNO_3 (100 mM). The suspension was left overnight. Prior to the acid-base titration, the suspension was further solvent-exchanged with 100 mM KNO_3 to ensure removal of any trace organic solvents. The pH of the suspension was adjusted to ~3 using 10-100 mM HCl solution. Then, 28 mM of aqueous NaOH solution was titrated in and at each increment, pH was recorded. This was repeated until the pH of the solution reaches ~10 and was repeated at least two times with different suspensions of Ti-MIL-125. The first-order derivatives of the titration curves were used to derive the pK_a values. These are shown in Figure 2B in the main text.

2.4 Dynamic Light Scattering

DLS measurements of Ti-MIL-125 crystallites were performed by combining the crystallite suspension in methanol to aqueous buffers used in the electrochemical studies (*vide infra*). Concentrations of Ti-MIL-125 were adjusted to be around 50 μM. Measurements were taken at fixed scattering angles of 90° (side scatter) and 175° (back scatter), with a range of 6 to 13 individual runs recorded at each angle at 25 °C. To ensure reliability in the data collection process,

a 2-minute equilibration time was provided prior to each measurement, followed by 10 seconds of acquisition time.

As described in the main text, **Ti-MIL-125-L** sedimented rapidly in the cuvette when added to pH 7-adjusted Tris buffer. This led to difficulty in measuring accurate crystal sizes using DLS (or electrochemical measurements; see below).

3 Physical Characterization of Ti-MIL-125-S, -M, and -L

3.1 N_2 Adsorption-Desorption Isotherms for three Ti-MIL-125 crystallite batches

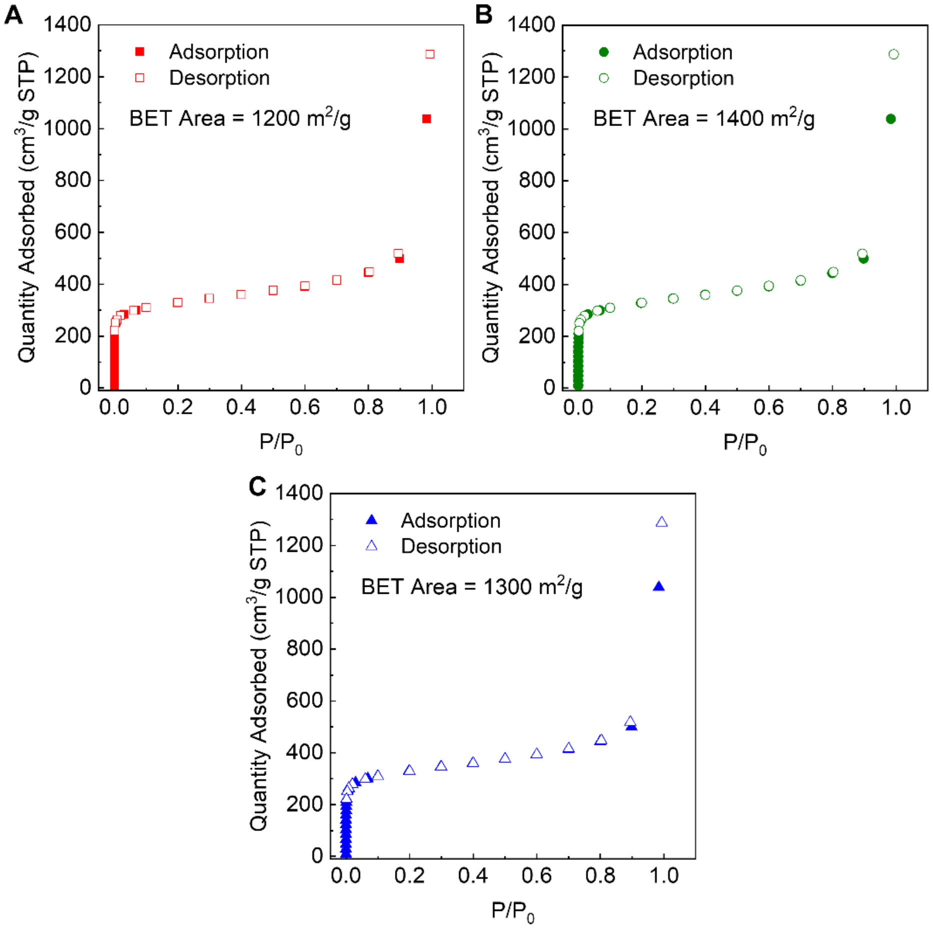

Figure S1. N_2-adsorption-desorption isotherms of **Ti-MIL-125-X** where **X** = **(A) S**, **(B) M**, **(C) L**. These figures are adopted from Altınçekiç, N.G.; Achemire, M.A.; Noh, H. Crystal-size-dependent Optical Properties of H-atoms on the Nodes of Ti-based Metal-organic Framework. Chemistry – An Asian Journal **2025**, 20 (5), e202401055.

3.2 PXRD Patterns of Ti-MIL-125 and Scherrer Equation

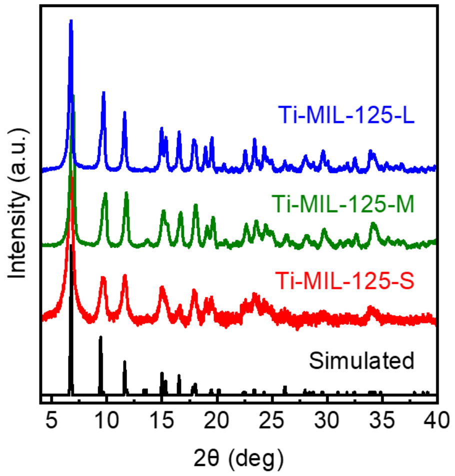

Figure S2. PXRD patterns of **Ti-MIL-125-S**, **-M**, and **-L**. The simulated PXRD pattern (black trace) is derived from the CIF in the reference.[3] This figure is adopted from Altınçekiç, N.G.; Achemire, M.A.; Noh, H. Crystal-size-dependent Optical Properties of H-atoms on the Nodes of Ti-based Metal-organic Framework. Chemistry – An Asian Journal **2025**, 20 (5), e202401055.

Crystallite sizes of the three batches were determined based on the first three peaks of the full-width-half-maximum (FWHM) from the PXRD patterns shown above. These FWHMs were then applied in the Scherrer equation (eq. S2) to estimate the average crystal size of Ti-MIL-125. In this equation, K, λ, θ, and β represent the Scherrer constant (assumed to be 0.9), incident light source wavelength (Cu Kα; 1.54 Å), the Bragg angle, and FWHM; the latter two are in radians.

$$d = \frac{K\lambda}{\beta cos\theta} \tag{S2}$$

From this equation, we estimated the average crystal sizes of **Ti-MIL-125-S**, **-M**, and **-L** to be 15, 22, and 33 nm, respectively.

3.3 STEM Images

Crystal sizes of 35 to 190 distinct crystallites were measured based on STEM images shown below in Figure S3. The average and the crystal size distributions are shown beside STEM images of each crystallite. These were used to derive the average crystal sizes of **Ti-MIL-125-S**, **-M**, and **-L** to be 19 ± 5, 50 ± 20, and 60 ± 20 nm, respectively. Here, the errors represent 1σ of the average values.

The average particle sizes from these images are larger than those estimated by PXRD patterns above, likely due to agglomeration, as Ti-MIL-125 crystallites don't have surfactants commonly used in conventional nanoparticles to avoid aggregation.

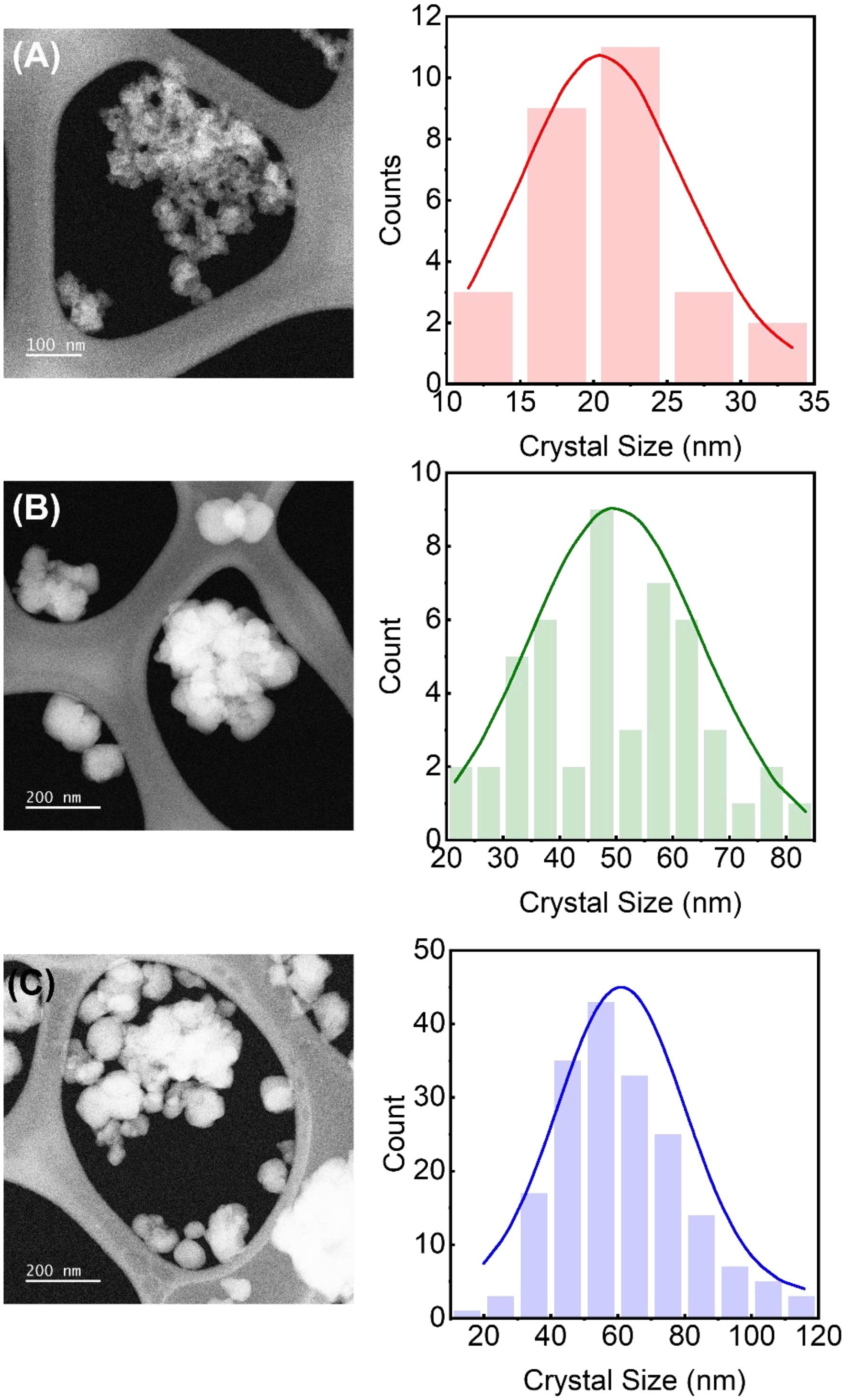

Figure S3. Representative STEM images and crystal size distributions of **Ti-MIL-125-X**, where **X** = (**A**) **S**, (**B**) **M**, (**C**) **L**.

3.4 TEM Images

Per the request of a reviewer, we have conducted high-resolution TEM imaging of **Ti-MIL-125-S** to examine whether the structural disorders can be observed. As shown below in Figure S4, we were unable to unequivocally determine the lattice packing and/or any surface structural changes.

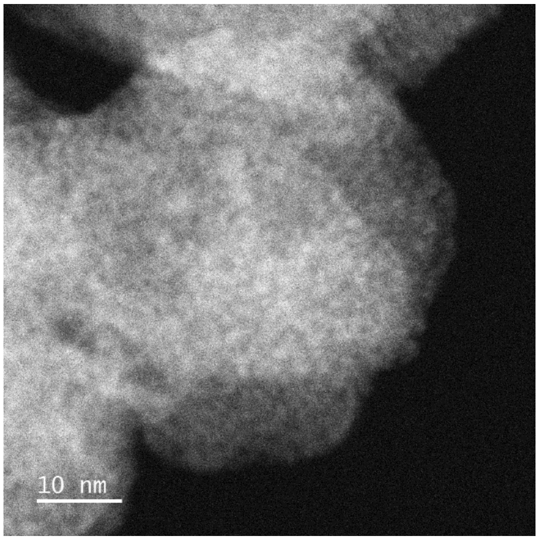

Figure S4. High-resolution TEM image of **Ti-MIL-125-S**.

3.5 DLS

As described in the main text, crystal sizes derived from DLS were, in general, significantly larger than any of the other techniques. This is likely due to crystal agglomeration, similar to that observed in the STEM images (see Figure S3). Depending on the electrolyte, the crystal size distribution widely varied in a trend that is difficult to understand; thus, we prefer to use PXRD patterns and STEM images as two primary tools to describe the crystal sizes of **Ti-MIL-125-S**, **-M**, and **-L**.

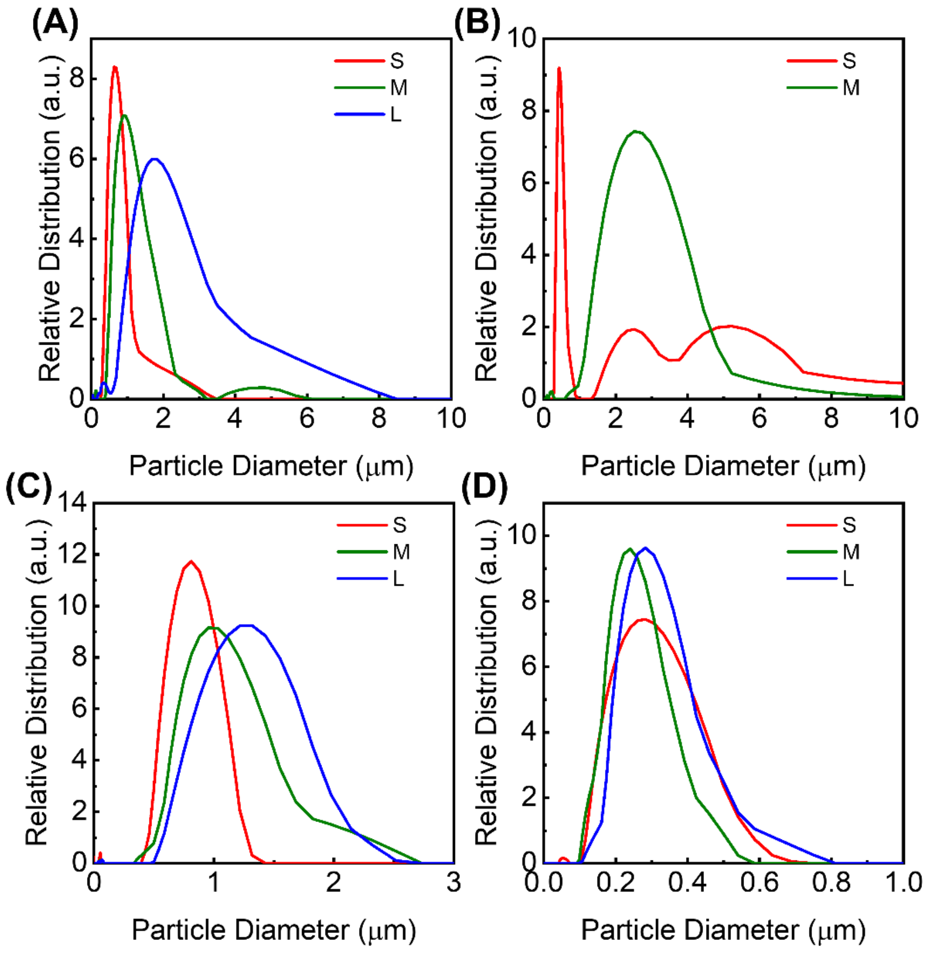

Figure S5. DLS results of **Ti-MIL-125-S**, **-M**, and **-L** in (**A**) pH 6-adjusted MES, (**B**) pH 7-adjusted Tris, (**C**) pH 8-adjusted Tris, or (**D**) pH 9-adjusted H_3BO_3, measured using the side detector.

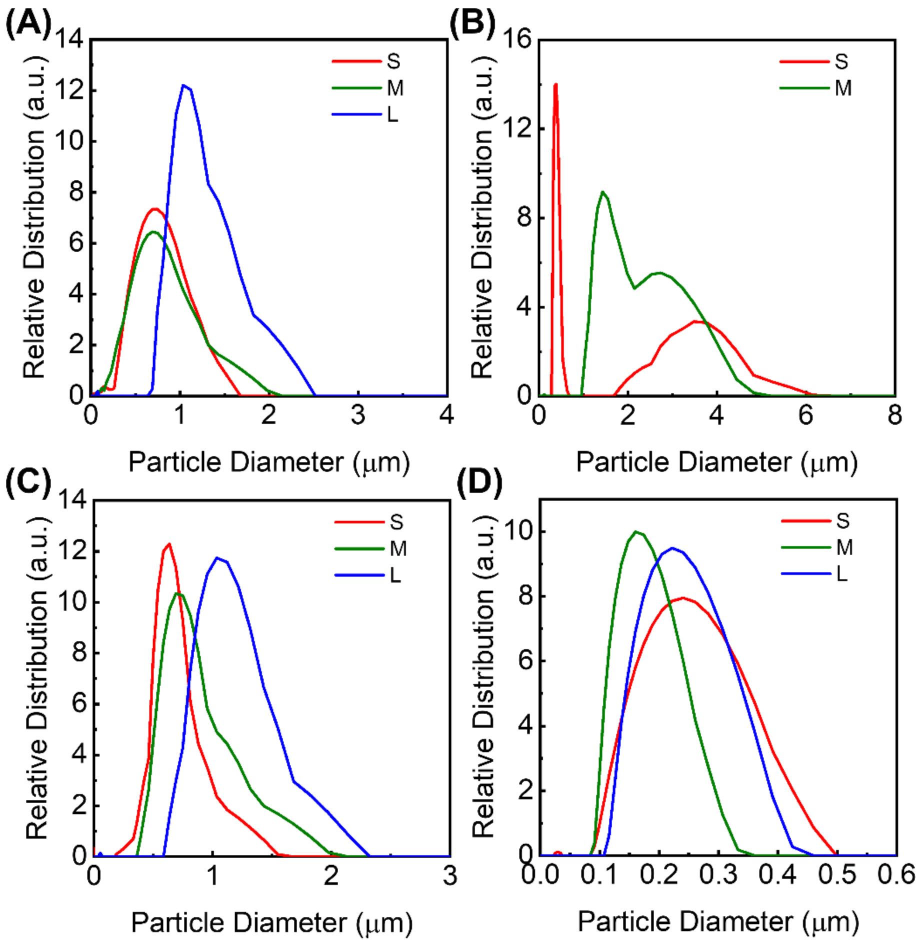

Figure S6. DLS results of **Ti-MIL-125-S**, **-M**, and **-L** in (**A**) pH 6-adjusted MES, (**B**) pH 7-adjusted Tris, (**C**) pH 8-adjusted Tris, or (**D**) pH 9-adjusted H_3BO_3, measured using the back detector.

3.6 UV-Visible Spectra

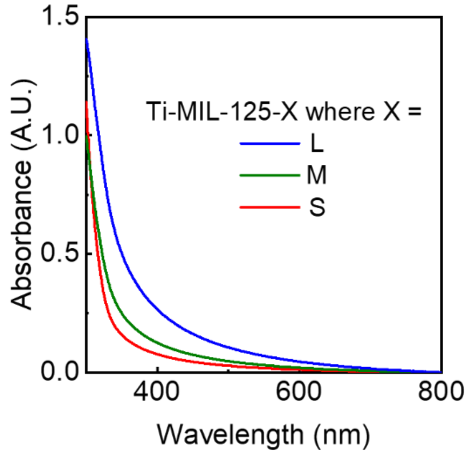

Figure S7. UV-visible spectra of the **Ti-MIL-125-S**, **-M**, and **-L**.

3.7 ^{1}H NMR Spectra

Concentrations of colloidal Ti-MIL-125 were derived using ^{1}H NMR, as described previously. Briefly, approximate concentrations of the three batches were determined by the ratio of BDC^{2-} linker to DMSO, which is an internal standard. Measurements were repeated at least twice. Approximate concentrations were found to be 5 ± 3, 29 ± 7, and 65 ± 1 mM for **Ti-MIL-125-S**, -**M**, and **-L**, respectively.

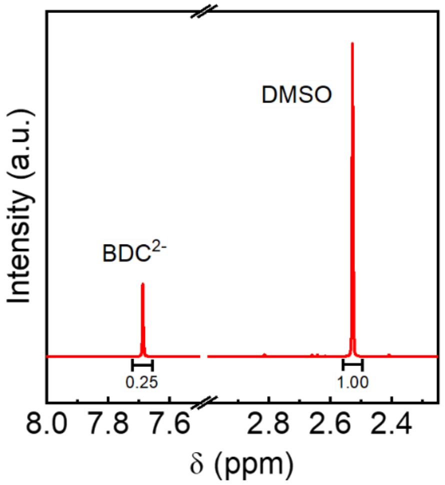

Figure S8. Representative ^{1}H NMR of digested Ti-MIL-125-L.

3.8 Zeta-Potential Measurement

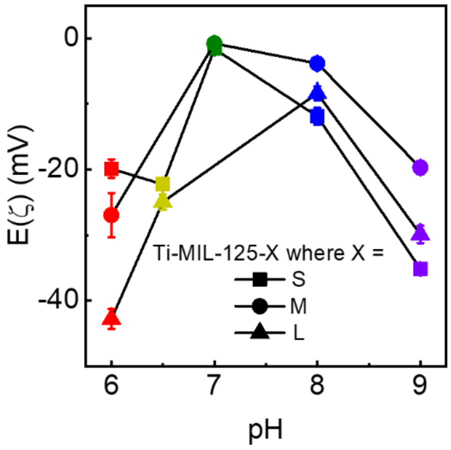

Figure S9. Zeta-potential of **Ti-MIL-125-S**, **-M**, and **-L** in various buffers and pH used for E_{OCP} measurements.

4 Open-Circuit Potentials of Ti-MIL-125-M and -L

E_{OCP} measurements of **Ti-MIL-125-M** and **-L** were conducted as reported previously for **Ti-MIL-125-S** in the following reference. As shown below, addition of photo-reduced, Ti^{3+}-MIL-125 led to a cathodic shift in E_{OCP} with an equilibration time between 300 to >1000 seconds for all crystallites. Here, we define equilibration as a change in E_{OCP} of <5 mV min^{-1}, following reports from us and others.

The average of the last 60 seconds of all measurements was used to determine the E_{OCP} at particular ratios of Ti^{3+} vs. Ti^{4+} and at electrolyte pH. 1σ represents the standard error of the average value. At least duplicate measurements at various ratios of Ti^{3+} vs. Ti^{4+} were conducted. Standard errors on the linear fits indicate 1σ from the linear regressions. As described previously, most linear fits had slopes between 50-70 mV/log($[Ti^{3+}]/[Ti^{4+}]$), which are generally considered 'Nernstian' (cf. [4,7-10]). The only exception is the linear fit of Ti-MIL-125-L at pH 6-adjusted MES buffer with the slope of 80(10) mV/log($[Ti^{3+}]/[Ti^{4+}]$). As shown in Figures 2D and 4 in the main text, this is the only data point that significantly deviates from the other data points. Removal of this data point does not alter the thermodynamics described in the main text.

In *Section 4.1*, we show representative E_{OCP} vs. time plots for all buffers employed for **Ti-MIL-125-M** and **-L**. Data points shown in black or green/blue are before and after the injection of Ti^{3+}-MIL-125, respectively. Right of these plots are compiled data of E_{OCP} vs. log($[Ti^{3+}]/[Ti^{4+}]$).

We have previously determined that E_{OCP} values are not obscured by (A) buffer concentration, (B) identity of working electrode, and (C) addition of formaldehyde or other oxidized products from methanol. We have also demonstrated that the duration used to calculate the average values at each reaction condition is independent of the resulting thermodynamics. We refer the readers to the following reference for more details.[4]

4.1 Results for Ti-MIL-125-M

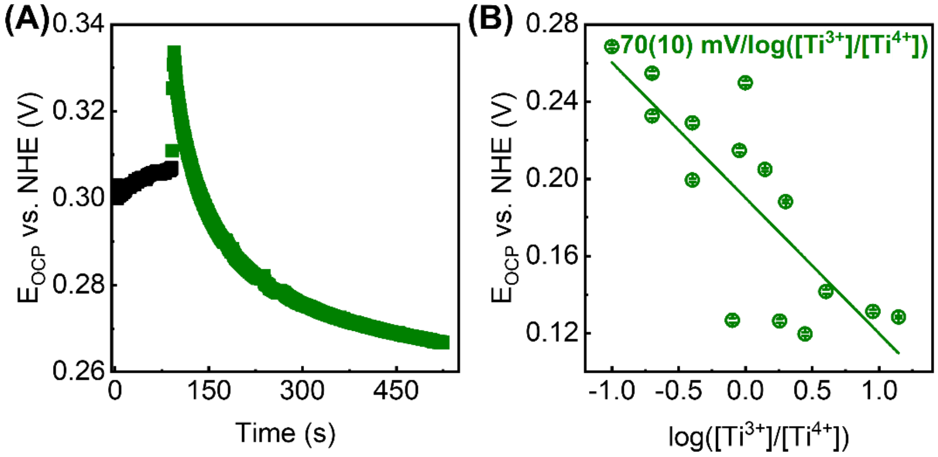

Figure S10. (**A**) Representative E_{OCP} vs. time plot and (**B**) E_{OCP} vs. log([Ti^{3+}]/[Ti^{4+}]) plot measured in pH 6-adjusted MES buffer using **Ti-MIL-125-M**.

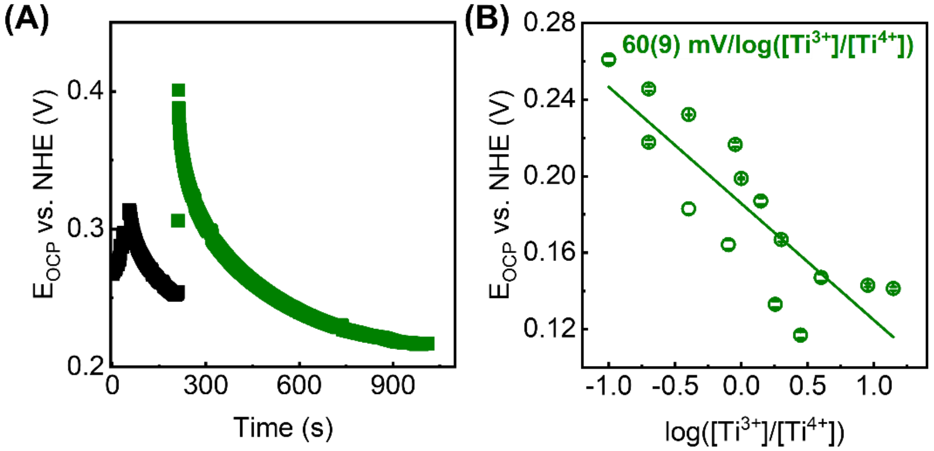

Figure S11. (**A**) Representative E_{OCP} vs. time plot and (**B**) E_{OCP} vs. log([Ti^{3+}]/[Ti^{4+}]) plot measured in pH 7-adjusted Tris buffer using **Ti-MIL-125-M**.

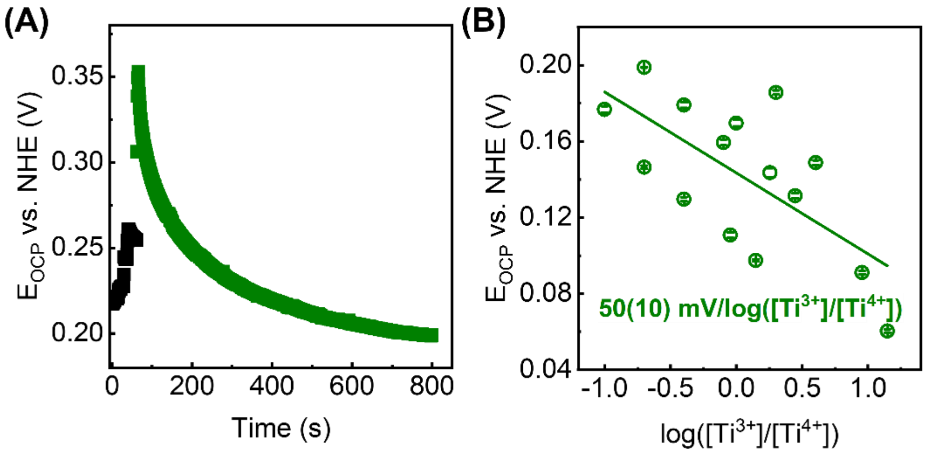

Figure S12. (**A**) Representative E_{OCP} vs. time plot and (**B**) E_{OCP} vs. log([Ti^{3+}]/[Ti^{4+}]) plot measured in pH 8-adjusted Tris buffer using **Ti-MIL-125-M**.

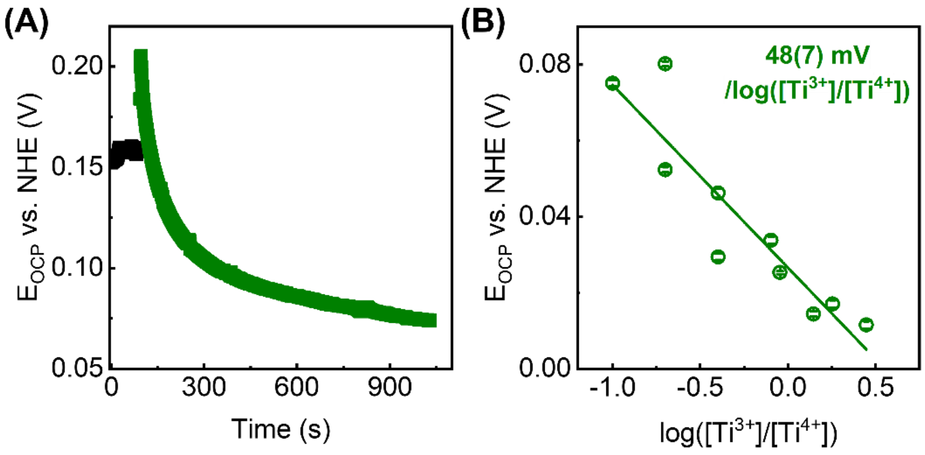

Figure S13. (**A**) Representative E_{OCP} vs. time plot and (**B**) E_{OCP} vs. log([Ti^{3+}]/[Ti^{4+}]) plot measured in pH 9-adjusted H_3BO_3 buffer using **Ti-MIL-125-M**.

4.2 Results for Ti-MIL-125-L

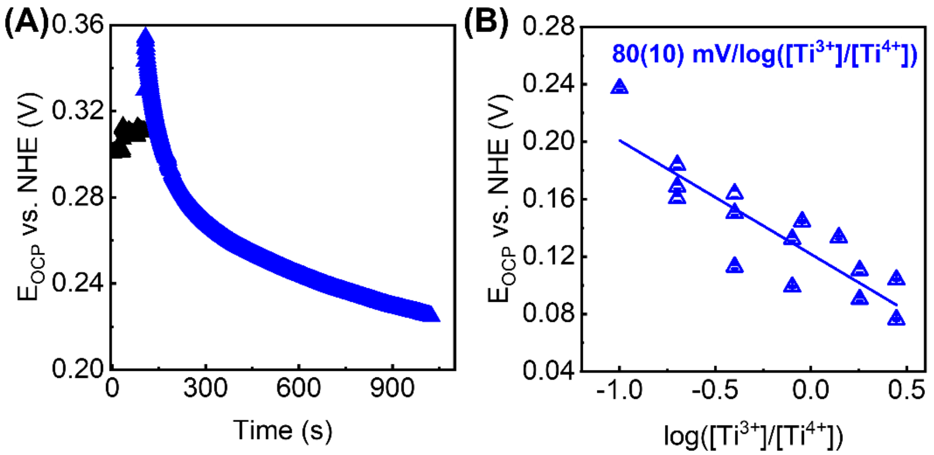

Figure S14. (**A**) Representative E_{OCP} vs. time plot and (**B**) E_{OCP} vs. log([Ti^{3+}]/[Ti^{4+}]) plot measured in pH 6-adjusted MES buffer using **Ti-MIL-125-L**.

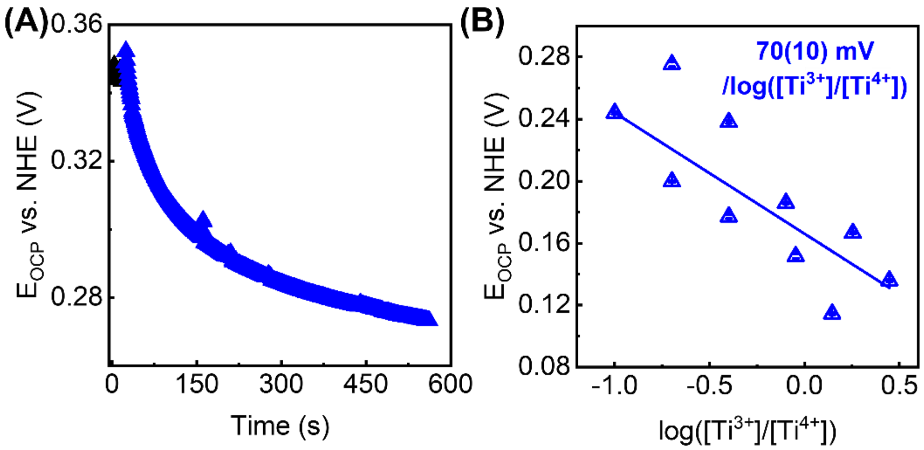

Figure S15. (**A**) Representative E_{OCP} vs. time plot and (**B**) E_{OCP} vs. log([Ti^{3+}]/[Ti^{4+}]) plot measured in pH 6.5-adjusted MES buffer using **Ti-MIL-125-L**.

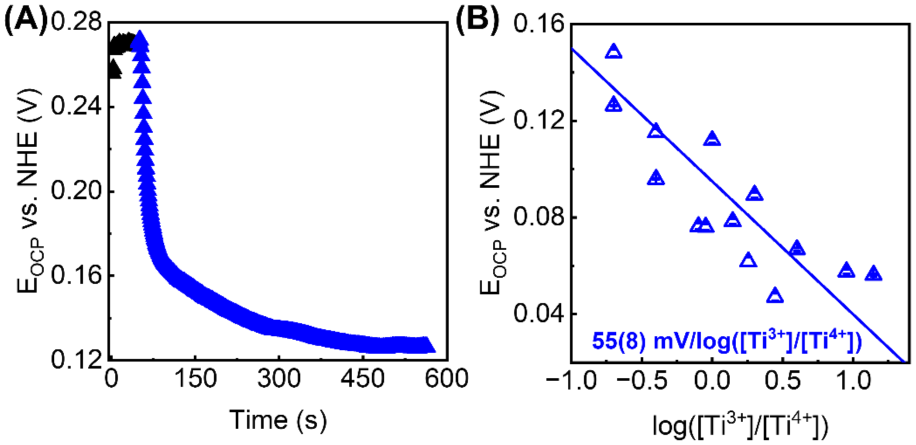

Figure S16. (**A**) Representative E_{OCP} vs. time plot and (**B**) E_{OCP} vs. log([Ti^{3+}]/[Ti^{4+}]) plot measured in pH 8-adjusted Tris buffer using **Ti-MIL-125-L**.

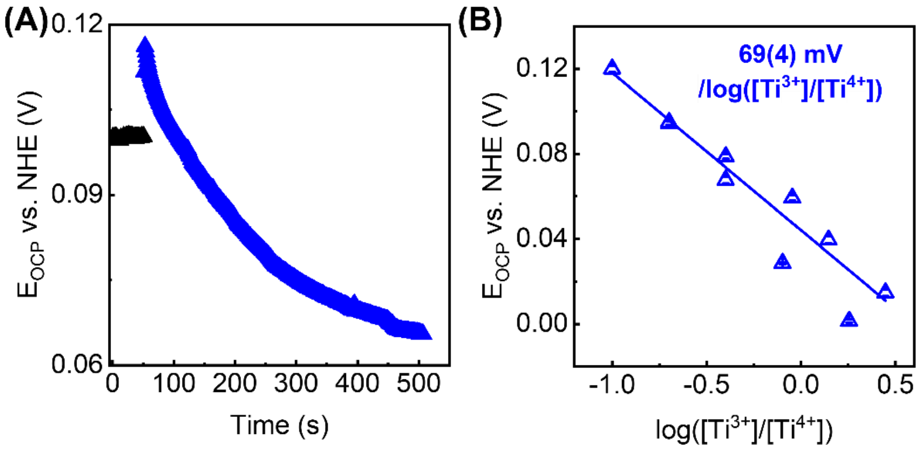

Figure S17. (**A**) Representative E_{OCP} vs. time plot and (**B**) E_{OCP} vs. log([Ti^{3+}]/[Ti^{4+}]) plot measured in pH 9-adjusted H_3BO_3 buffer using **Ti-MIL-125-L**.

5 Details on the Computational Calculations

5.1 Ti_8 Nodes with H-atoms and their calculated Ti^{3+}O–H BDE/Bond Distance Changes

PBE0 functional[11] and the def2-SVP basis set in Q-Chem 6.3[12] was employed to simulate the O−H bond dissociation energy at missing-linker defect sites of Ti-MIL-125, following our earlier work. We note that as described in our previous work, arbitrarily high translational enthalpy of H-atom prior to the node-binding precludes accurate measurement of bond dissociation free energy (BDFE). Our previous works have demonstrated multiple times that computed BDE is quantitatively similar to experimentally measured BDFEs.[4,13]

To the geometry-optimized, pristine node of Ti-MIL-125, we have replaced one formate unit with an −OH/−OH_2 pair. This structure is shown as Figure 3A in the main text and is reproduced below as Figure S17 with labels used for Table S1.

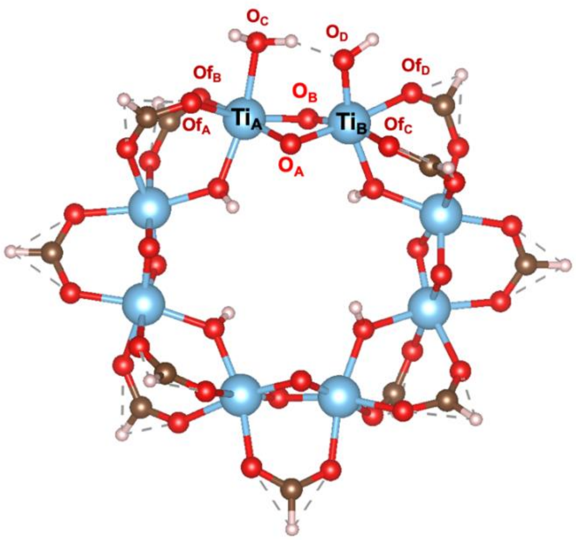

Figure S18. Ti_8 node with missing-linker defect sites and labels on Ti and O-atoms.

Table S9 below describes the change in bond distance upon the addition of $1H^+/1e^-$ or $2H^+/2e^-$. The proton topologies labeled below correspond to that shown in Figure 3 in the main text.

Table S9. Ti-O bond distances for pristine vs. reduced Ti_8 nodes.

**Atom Indices**			**Redox States**	**Bond Distance (Å)**		**Bond Distance Change upon Reduction (Å)**	
**Ti_1**	**Ti_2**	**O**		**Ti_1–O**	**Ti_2–O**	**Ti_1–O**	**Ti_2–O**
Proton Topology B:							
Ti_A	Ti_B	O_A	Oxidized	1.87	1.76	–	–
			Reduced	2.1	1.95	0.23	0.19
		O_B	Oxidized	1.72	1.94	–	–
			Reduced	1.88	1.74	0.16	–0.2
	–	O_{fA}	Oxidized	2.17	–	–	–
			Reduced	2.08	–	–0.09	–
		O_C	Oxidized	2.06	–	–	–
			Reduced	2.15	–	0.09	–
		O_{fB}	Oxidized	1.98	–	–	–
			Reduced	2.04			–

Continued to the next page.

Ti_B –		O_D	Oxidized	1.87	–	–	–
			Reduced	1.87	–	0	–
		O_{fC}	Oxidized	1.98	–	–	–
			Reduced	2.13	–	0.15	–
		O_{fD}	Oxidized	2.17	–	–	–
			Reduced	2.04	–	–0.13	–
Proton Topology C:							
	Ti_B	O_A	Oxidized	1.87	1.76	–	–
			Reduced	1.82	2.82	–0.05	1.06
		O_B	Oxidized	1.72	1.94	–	–
			Reduced	1.69	2.02	–0.03	0.08
Ti_A	–	O_{fA}	Oxidized	2.17	–	–	–
			Reduced	2.24	–	0.07	–
		O_C	Oxidized	2.06	–	–	–
			Reduced	2.15	–	0.09	–
		O_{fB}	Oxidized	1.98	–	–	–
			Reduced	2.04	–	–0.06	–
Ti_B	–	O_D	Oxidized	1.87	–	–	–
			Reduced	2.28	–	0.41	–
		O_{fC}	Oxidized	1.98	–	–	–
			Reduced	2.09	–	0.11	–
		O_{fD}	Oxidized	2.17	–	–	–
			Reduced	2.15	–	–0.02	–

Continued to the next page.

Proton Topology D:							
Ti_A	Ti_B	O_A	Oxidized	1.87	1.76	–	–
			Reduced	2.06	2.04	0.19	0.28
		O_B	Oxidized	1.72	1.94	–	–
			Reduced	1.84	1.79	0.12	–0.15
		O_{fA}	Oxidized	2.17	–	–	–
			Reduced	2.12	–	–0.05	–
		O_C	Oxidized	2.06	–	–	–
			Reduced	2.19	–	0.13	–
		O_{fB}	Oxidized	1.98	–	–	–
			Reduced	2.1	–	0.12	–
Ti_B	–	O_D	Oxidized	1.87	–	–	–
			Reduced	2.25	–	0.38	–
		O_{fC}	Oxidized	1.98	–	–	–
			Reduced	2.18	–	0.2	–
		O_{fD}	Oxidized	2.17	–	–	–
			Reduced	2.1	–	–0.07	–

5.2 XYZ Coordinates of Computational Models

The XYZ coordinates of pristine and reduced Ti_8 nodes (formate-terminated, replaced one unit with an −OH/−OH₂ pair) in various proton topologies can be found below. The labels of individual proton topologies are listed in the main text.

Original Ti_8 Node:

C	-2.88640	3.80514	-2.39632
C	-4.02171	4.05642	1.17243
O	-0.90960	5.98935	0.65545
O	-5.57507	1.31173	-1.13536
O	-2.19583	4.39224	-1.51779
O	-4.39129	3.13387	0.40756
O	-2.86694	4.50963	1.28224
O	-3.41265	2.69140	-2.27622
O	-1.81329	2.12443	-0.04570
Ti	-1.11612	4.00761	0.09984
Ti	-3.64433	1.50661	-0.53766
C	3.86189	4.20500	-0.95474
C	3.81001	2.68085	2.46871
O	5.70539	0.99771	-0.78256
O	1.39592	5.58108	1.55003
O	0.44831	4.05795	-0.60933
O	-0.19033	3.43811	1.63137
O	4.11144	3.01377	-1.26816
O	2.64658	3.17111	2.48694
O	4.37708	2.19037	1.48342
O	3.15390	4.59074	-0.00836
O	1.96225	1.99114	0.13534
Ti	3.73733	1.28563	-0.30672
Ti	1.43318	3.77652	1.04623
C	-6.20489	0.22997	-1.08311
C	-3.81545	-4.07073	-1.10317
C	-3.83799	-2.81822	2.34469
O	-5.73104	-0.87896	-0.74304
O	-1.23288	-5.61027	1.35755
O	-3.24417	-0.04375	-1.52800
O	-3.82869	0.37163	0.76310
O	-4.09840	-2.86715	-1.35113
O	-2.66584	-3.28581	2.32937
O	-4.38322	-2.20617	1.41608
O	-3.06671	-4.47958	-0.20367
O	-1.94301	-1.85989	0.09779
Ti	-3.76756	-1.18619	-0.32004
Ti	-1.40526	-3.66050	0.84164
C	-0.20128	-6.28867	1.14573
C	6.16728	-0.08853	-1.19783
C	2.81942	-3.69657	-2.47430
C	4.05989	-3.91111	1.04315
O	0.86446	-5.86131	0.64279
O	5.53351	-1.16626	-1.29906
O	-0.41276	-3.94611	-0.70182
O	3.19844	0.19506	-1.56315
O	0.17086	-3.33216	1.54951
O	3.82845	-0.31715	0.69647
O	2.20272	-4.30200	-1.55328
O	4.42470	-3.03651	0.21915
O	2.90112	-4.33100	1.21304
O	3.30282	-2.56034	-2.39148
O	1.82299	-2.04129	-0.08677
Ti	1.16922	-3.94002	0.09022
Ti	3.62668	-1.39168	-0.65644
H	-4.81128	4.51455	1.80520
H	4.36190	2.70692	3.43087
H	-4.41820	-2.98018	3.27604
H	4.85774	-4.35531	1.67500
H	-3.02643	4.35060	-3.35230
H	4.33477	4.98059	-1.59384
H	-7.27625	0.25622	-1.36312
H	-4.29603	-4.82454	-1.76120
H	-0.23210	-7.35873	1.42849
H	7.23148	-0.10200	-1.50539
H	2.93154	-4.24774	-3.43044
H	-1.45467	1.62697	0.70369
H	1.50282	-1.54772	0.68209
H	1.35115	1.85610	-0.60376
H	-1.32063	-1.60417	-0.59823
H	-1.56135	6.15201	1.35293
H	0.02706	6.04003	1.06224
H	2.15875	6.04753	1.18040

Proton Topology A:

C	-3.17350	3.73020	-2.52004
C	-4.04739	3.94662	1.18190
O	-0.99499	5.95192	0.30834
O	-5.85852	1.24139	-0.96436
O	-2.39847	4.35881	-1.77457
O	-4.53639	3.07444	0.44648
O	-2.85001	4.33743	1.19603
O	-3.72579	2.63482	-2.25497
O	-2.00503	1.96856	-0.21164
Ti	-1.30346	3.86742	-0.11870
Ti	-3.84249	1.41099	-0.66528
C	4.18268	4.20326	-0.79604
C	3.57102	2.67664	2.62147
O	6.00893	0.93075	-0.48709
O	1.29748	5.50646	1.34583
O	0.47348	3.83837	-0.75248
O	-0.16077	3.22966	1.52793
O	4.58852	3.04626	-0.98625
O	2.45330	3.19419	2.47085
O	4.29232	2.19140	1.71030
O	3.24918	4.56496	-0.03150
O	2.21209	1.96452	0.03150
Ti	4.05396	1.24957	-0.03288
Ti	1.49813	3.77266	0.65827
C	-6.50179	0.19866	-0.71794
C	-4.03296	-4.02160	-1.11463
C	-3.63214	-2.98519	2.47339
O	-6.01676	-0.88180	-0.29887
O	-1.16873	-5.58524	1.00727
O	-3.61143	-0.15978	-1.45341
O	-3.96315	0.38471	0.86265
O	-4.51674	-2.87805	-1.13336
O	-2.51788	-3.48458	2.27708
O	-4.28406	-2.28834	1.64662
O	-3.06284	-4.41512	-0.41698
O	-2.13669	-1.84215	0.02121
Ti	-4.05289	-1.18033	0.02350
Ti	-1.42612	-3.64440	0.46192
C	-0.07564	-6.18889	0.91049
C	6.44534	-0.12754	-0.99686
C	2.95338	-3.68485	-2.43122
C	4.14573	-3.76551	1.06199
O	1.01547	-5.68020	0.56456
O	5.76686	-1.14662	-1.26243
O	-0.30023	-3.75903	-0.85346
O	3.52869	0.28818	-1.52870
O	0.14178	-3.22061	1.44185
O	4.00694	-0.35505	0.73796
O	2.31119	-4.25159	-1.53622
O	4.57776	-2.99881	0.18666
O	2.93882	-4.04798	1.27934
O	3.44802	-2.52617	-2.36351
O	1.96199	-1.86586	-0.16137
Ti	1.27335	-3.69402	0.21251
Ti	3.79663	-1.31313	-0.82441
H	-4.73140	4.44515	1.90241
H	3.98962	2.64098	3.64931
H	-4.11843	-3.15701	3.45595
H	4.88616	-4.26230	1.72353
H	-3.40448	4.17596	-3.50911
H	4.69830	5.01694	-1.34944
H	-7.59673	0.22408	-0.88254
H	-4.50062	-4.78366	-1.77301
H	-0.07354	-7.26982	1.15304
H	7.52620	-0.16513	-1.23536
H	3.11930	-4.23297	-3.38148
H	-1.66437	1.48376	0.55443
H	1.69103	-1.25902	0.54237
H	1.74303	1.72044	-0.77905
H	-1.68871	-1.44710	-0.74021
H	-1.64305	6.16684	0.99146
H	-0.09025	5.99946	0.74034
H	2.08258	6.05908	1.25418
H	-0.27472	3.47203	2.45404

Proton Topology B:

C	-3.54932	3.29322	2.50008
C	-3.97881	4.28382	-1.04377
O	-5.96349	1.25269	-0.18631
O	-0.99015	5.86417	0.91772
O	-4.18341	2.50002	1.76760
O	-2.91554	4.64102	-0.47913
O	-4.51000	3.16149	-0.98636
O	-2.45594	3.82666	2.23221
O	-1.96788	2.06760	0.12318
Ti	-3.84832	1.33209	0.12263
Ti	-1.31262	3.89410	0.49877
C	-4.34683	-3.70152	0.92215
C	-3.01876	-3.58612	-2.56858
O	-1.26216	-5.79691	0.52265
O	-5.84275	-1.19242	-1.41265
O	-3.80691	-0.18053	0.87799
O	-3.52364	0.48703	-1.46286
O	-3.18292	-4.08060	1.13626
O	-3.53308	-2.44684	-2.53821
O	-2.42085	-4.14409	-1.62172
O	-4.70657	-2.85286	0.06526
O	-1.86400	-1.90145	-0.10653
Ti	-1.38206	-3.79713	0.10527
Ti	-3.62998	-1.22328	-0.84778
C	0.12654	6.41948	0.81251
C	4.24543	3.68889	1.12481
C	3.06897	3.62417	-2.37652
O	1.20391	5.85325	0.51037
O	5.62888	0.92387	-1.19880
O	0.23270	3.49989	1.50484
O	-0.16575	3.91204	-0.82881
O	3.08125	4.13013	1.28584
O	3.45245	2.42978	-2.27064
O	2.47391	4.27381	-1.50363
O	4.60252	2.81773	0.31155
O	1.93950	1.94160	-0.02728
Ti	1.37447	3.85281	0.23758
Ti	3.68847	1.21997	-0.69034
C	6.23780	-0.15232	-0.99963
C	-0.23298	-6.34074	0.98296
C	3.57562	-3.13420	2.47549
C	3.68663	-4.27923	-1.05011
O	5.73553	-1.20180	-0.53488
O	0.86786	-5.77511	1.18988
O	3.85689	0.17272	0.79859
O	-0.35177	-3.41963	1.53500
O	3.26586	-0.34246	-1.46969
O	0.20337	-3.92211	-0.75132
O	4.17944	-2.47774	1.60043
O	2.84293	-4.66741	-0.21062
O	4.10036	-3.11263	-1.20312
O	2.41991	-3.59949	2.37249
O	1.88905	-2.02111	0.17685
Ti	3.76971	-1.39448	-0.07495
Ti	1.22000	-3.83500	0.74132
H	-4.48788	5.06326	-1.65122
H	-3.11051	-4.16170	-3.51346
H	3.29120	4.12947	-3.33915
H	4.11546	-5.05376	-1.72091
H	-4.00948	3.54478	3.47894
H	-5.14843	-4.15841	1.54027
H	0.16910	7.51009	1.00250
H	5.02934	4.12962	1.77598
H	7.31428	-0.17925	-1.25831
H	-0.29644	-7.41865	1.23030
H	4.11375	-3.32318	3.42684
H	-1.52083	1.70914	-0.65682
H	1.33887	-1.69469	-0.55017
H	-1.70207	-1.43325	0.72701
H	1.60836	1.40305	0.70566
H	-6.07233	2.01261	-0.78306
H	-6.17381	0.44385	-0.69334
H	-5.98299	-1.99113	-0.86004
H	-5.87134	-1.48286	-2.33467

Proton Topology C:

C	-3.94759	-3.31248	2.30243
C	-4.74568	-2.98290	-1.29792
O	-2.22380	-5.64161	-0.77308
O	-5.93984	-0.18949	1.14976
O	-3.37946	-4.03737	1.47323
O	-5.02892	-2.12128	-0.44592
O	-3.64598	-3.57480	-1.43155
O	-4.09258	-2.06152	2.21976
O	-2.30392	-1.55362	0.19782
Ti	-2.02828	-3.57448	-0.06476
Ti	-3.99615	-0.72618	0.75523
C	3.22495	-4.77625	1.31687
C	3.24853	-3.81520	-2.23908
O	5.65651	-2.00831	0.64663
O	0.49261	-6.03831	-1.21150
O	-0.48667	-4.01844	0.83266
O	-0.68644	-3.28770	-1.60036
O	3.72396	-3.64095	1.42103
O	2.05254	-4.17669	-2.17795
O	3.85503	-3.14415	-1.37777
O	2.32457	-5.12790	0.51236
O	1.71548	-2.15625	0.06128
Ti	3.64358	-1.91138	0.24613
Ti	0.81648	-3.96640	-0.39241
C	-6.38125	0.96797	0.99459
C	-3.14781	4.68371	1.30471
C	-3.18310	3.63421	-2.27819
O	-5.71619	1.96426	0.61276
O	-0.22532	5.72131	-1.05262
O	-3.45050	0.74175	1.59864
O	-3.97491	0.40328	-0.72409
O	-3.79791	3.63089	1.40198
O	-1.96934	3.86898	-2.18592
O	-3.89832	3.10160	-1.38914
O	-2.19210	4.90670	0.51561
O	-1.73837	2.20699	0.08318
Ti	-3.75229	1.89628	0.19501
Ti	-0.77101	3.85502	-0.44560
C	0.95700	6.13192	-1.05364
C	6.33701	-1.01760	0.99058
C	3.72138	3.27750	2.20344
C	4.69547	2.96041	-1.34600
O	1.97037	5.45360	-0.76319
O	5.91643	0.16063	1.10600
O	0.44126	3.81490	0.80707
O	3.42104	-0.71821	1.59192
O	0.64576	3.16368	-1.49425
O	3.95563	-0.43481	-0.73552
O	3.13115	3.91310	1.31649
O	5.02355	2.13829	-0.47093
O	3.56420	3.48386	-1.49410
O	3.99567	2.04954	2.16938
O	2.29572	1.55748	0.05449
Ti	1.90663	3.47171	-0.33371
Ti	4.02461	0.70778	0.68065
H	-5.54950	-3.26554	-2.01245
H	3.83087	-4.12159	-3.13429
H	-3.69780	3.91578	-3.22031
H	5.47640	3.27132	-2.07141
H	-4.37312	-3.79236	3.20810
H	3.61574	-5.56045	2.00023
H	-7.45370	1.13960	1.21246
H	-3.43091	5.52336	1.97416
H	1.12415	7.18980	-1.33692
H	7.40804	-1.18878	1.21593
H	4.04409	3.82974	3.11019
H	-1.90291	-1.01671	-0.49783
H	1.89510	0.99121	-0.61993
H	1.24195	-1.70624	0.77389
H	-1.32408	1.76667	0.83859
H	-2.74816	-5.51129	-1.57601
H	-1.35126	-5.97315	-1.05066
H	1.10772	-6.49249	-0.61066
H	1.00061	-5.87067	-2.02587
H	-0.56478	-2.45842	-2.07239

6 References

1. Fabrizio, K.; Gormley, E. L.; Davenport, A. M.; Hendon, C. H.; Brozek, C. K., Gram-scale synthesis of MIL-125 nanoparticles and their solution processability. *Chem. Sci.* **2023,** *14* (33), 8946-8955.
2. Howarth, A. J.; Peters, A. W.; Vermeulen, N. A.; Wang, T. C.; Hupp, J. T.; Farha, O. K., Best Practices for the Synthesis, Activation, and Characterization of Metal–Organic Frameworks. *Chem. Mater.* **2017,** *29* (1), 26-39.
3. Dan-Hardi, M.; Serre, C.; Frot, T.; Rozes, L.; Maurin, G.; Sanchez, C.; Férey, G., A New Photoactive Crystalline Highly Porous Titanium(IV) Dicarboxylate. *J. Am. Chem. Soc.* **2009,** *131* (31), 10857-10859.
4. Altınçekiç, N. G.; Lander, C. W.; Roslend, A.; Yu, J.; Shao, Y.; Noh, H., Electrochemically Determined and Structurally Justified Thermochemistry of H atom Transfer on Ti-Oxo Nodes of the Colloidal Metal–Organic Framework Ti-MIL-125. *J. Am. Chem. Soc.* **2024,** *146* (49), 33485–33498.
5. Altınçekiç, N. G.; Achemire, M. A.; Noh, H., Crystal-size-dependent Optical Properties of H-atoms on the Nodes of Ti-based Metal-organic Framework. *Chem. Asian J.* **2025,** *20* (5), e202401055.
6. Klet, R. C.; Liu, Y.; Wang, T. C.; Hupp, J. T.; Farha, O. K., Evaluation of Brønsted acidity and proton topology in Zr- and Hf-based metal–organic frameworks using potentiometric acid–base titration. *J. Mater. Chem. A* **2016,** *4* (4), 1479-1485.
7. Noh, H.; Mayer, J. M., Medium-Independent Hydrogen Atom Binding Isotherms of Nickel Oxide Electrodes. *Chem* **2022,** *8* (12), 3324-3345.
8. Nedzbala, H. S.; Westbroek, D.; Margavio, H. R. M.; Yang, H.; Noh, H.; Magpantay, S. V.; Donley, C. L.; Kumbhar, A. S.; Parsons, G. N.; Mayer, J. M., Photoelectrochemical Proton-Coupled Electron Transfer of TiO_2 Thin Films on Silicon. *J. Am. Chem. Soc.* **2024,** *146* (15), 10559-10572.
9. Wise, C. F.; Agarwal, R. G.; Mayer, J. M., Determining Proton-Coupled Standard Potentials and X–H Bond Dissociation Free Energies in Nonaqueous Solvents Using Open-Circuit Potential Measurements. *J. Am. Chem. Soc.* **2020,** *142* (24), 10681-10691.
10. Jackson, M. N.; Pegis, M. L.; Surendranath, Y., Graphite-Conjugated Acids Reveal a Molecular Framework for Proton-Coupled Electron Transfer at Electrode Surfaces. *ACS Cent. Sci.* **2019,** *5* (5), 831-841.
11. Perdew, J. P.; Burke, K.; Ernzerhof, M., Generalized Gradient Approximation Made Simple. *Phys. Rev. Lett.* **1996,** *77* (18), 3865-3868.
12. Epifanovsky, E.; Gilbert, A. T. B.; Feng, X.; Lee, J.; Mao, Y.; Mardirossian, N.; Pokhilko, P.; White, A. F.; Coons, M. P.; Dempwolff, A. L.; Gan, Z.; Hait, D.; Horn, P. R.; Jacobson, L. D.; Kaliman, I.; Kussmann, J.; Lange, A. W.; Lao, K. U.; Levine, D. S.; Liu, J.; McKenzie, S. C.; Morrison, A. F.; Nanda, K. D.; Plasser, F.; Rehn, D. R.; Vidal, M. L.; You, Z.-Q.; Zhu, Y.; Alam, B.; Albrecht, B. J.; Aldossary, A.; Alguire, E.; Andersen, J. H.; Athavale, V.; Barton, D.; Begam, K.; Behn, A.; Bellonzi, N.; Bernard, Y. A.; Berquist, E. J.; Burton, H. G. A.; Carreras, A.; Carter-Fenk, K.; Chakraborty, R.; Chien, A. D.; Closser, K. D.; Cofer-Shabica, V.; Dasgupta, S.; de Wergifosse, M.; Deng, J.; Diedenhofen, M.; Do, H.; Ehlert, S.; Fang, P.-T.; Fatehi, S.; Feng, Q.; Friedhoff, T.; Gayvert, J.; Ge, Q.; Gidofalvi, G.; Goldey, M.; Gomes, J.; González-Espinoza, C. E.; Gulania, S.; Gunina, A. O.; Hanson-Heine, M. W. D.; Harbach, P. H. P.; Hauser, A.; Herbst, M. F.; Hernández Vera, M.; Hodecker, M.; Holden, Z. C.; Houck, S.; Huang, X.; Hui, K.; Huynh, B. C.;

Ivanov, M.; Jász, Á.; Ji, H.; Jiang, H.; Kaduk, B.; Kähler, S.; Khistyaev, K.; Kim, J.; Kis, G.; Klunzinger, P.; Koczor-Benda, Z.; Koh, J. H.; Kosenkov, D.; Koulias, L.; Kowalczyk, T.; Krauter, C. M.; Kue, K.; Kunitsa, A.; Kus, T.; Ladjánszki, I.; Landau, A.; Lawler, K. V.; Lefrancois, D.; Lehtola, S.; Li, R. R.; Li, Y.-P.; Liang, J.; Liebenthal, M.; Lin, H.-H.; Lin, Y.-S.; Liu, F.; Liu, K.-Y.; Loipersberger, M.; Luenser, A.; Manjanath, A.; Manohar, P.; Mansoor, E.; Manzer, S. F.; Mao, S.-P.; Marenich, A. V.; Markovich, T.; Mason, S.; Maurer, S. A.; McLaughlin, P. F.; Menger, M. F. S. J.; Mewes, J.-M.; Mewes, S. A.; Morgante, P.; Mullinax, J. W.; Oosterbaan, K. J.; Paran, G.; Paul, A. C.; Paul, S. K.; Pavošević, F.; Pei, Z.; Prager, S.; Proynov, E. I.; Rák, Á.; Ramos-Cordoba, E.; Rana, B.; Rask, A. E.; Rettig, A.; Richard, R. M.; Rob, F.; Rossomme, E.; Scheele, T.; Scheurer, M.; Schneider, M.; Sergueev, N.; Sharada, S. M.; Skomorowski, W.; Small, D. W.; Stein, C. J.; Su, Y.-C.; Sundstrom, E. J.; Tao, Z.; Thirman, J.; Tornai, G. J.; Tsuchimochi, T.; Tubman, N. M.; Veccham, S. P.; Vydrov, O.; Wenzel, J.; Witte, J.; Yamada, A.; Yao, K.; Yeganeh, S.; Yost, S. R.; Zech, A.; Zhang, I. Y.; Zhang, X.; Zhang, Y.; Zuev, D.; Aspuru-Guzik, A.; Bell, A. T.; Besley, N. A.; Bravaya, K. B.; Brooks, B. R.; Casanova, D.; Chai, J.-D.; Coriani, S.; Cramer, C. J.; Cserey, G.; DePrince, A. E., III; DiStasio, R. A., Jr.; Dreuw, A.; Dunietz, B. D.; Furlani, T. R.; Goddard, W. A., III; Hammes-Schiffer, S.; Head-Gordon, T.; Hehre, W. J.; Hsu, C.-P.; Jagau, T.-C.; Jung, Y.; Klamt, A.; Kong, J.; Lambrecht, D. S.; Liang, W.; Mayhall, N. J.; McCurdy, C. W.; Neaton, J. B.; Ochsenfeld, C.; Parkhill, J. A.; Peverati, R.; Rassolov, V. A.; Shao, Y.; Slipchenko, L. V.; Stauch, T.; Steele, R. P.; Subotnik, J. E.; Thom, A. J. W.; Tkatchenko, A.; Truhlar, D. G.; Van Voorhis, T.; Wesolowski, T. A.; Whaley, K. B.; Woodcock, H. L., III; Zimmerman, P. M.; Faraji, S.; Gill, P. M. W.; Head-Gordon, M.; Herbert, J. M.; Krylov, A. I., Software for the frontiers of quantum chemistry: An overview of developments in the Q-Chem 5 package. *J. Chem. Phys.* **2021,** *155* (8).

13. Ingram, Z. J.; Lander, C. W.; Oliver, M. C.; Altınçekiç, N. G.; Huang, L.; Shao, Y.; Noh, H., Hydrogen-Atom Binding Energy of Structurally Well-defined Cerium Oxide Nodes at the Metal−Organic Framework-Liquid Interfaces. *J. Phys. Chem. C* **2024,** *128* (23), 9556–9565.
